# Critical evaluation of the theory and practice of feed-forward neural networks for genomic prediction

**DOI:** 10.1093/g3journal/jkaf314

**Published:** 2025-12-24

**Authors:** Aaron Kusmec, Karlene L Negus, Jianming Yu

**Affiliations:** Department of Agronomy, Kansas State University, Manhattan, KS 66506-0110, United States; Department of Agronomy, Iowa State University, Ames, IA 50011-1051, United States; Department of Agronomy, Iowa State University, Ames, IA 50011-1051, United States

**Keywords:** genomic prediction, phenotypic prediction, deep learning, linear models

## Abstract

Genomic prediction (GP) has catalyzed increased rates of genetic gain in animal and plant breeding. Recently, deep learning (DL) has been explored to increase GP accuracy by incorporating diverse data types and learning complex, non-linear patterns in datasets. However, DL consistently fails to significantly improve prediction accuracy over gold standard genomic BLUP (gBLUP) models. In this study, we first review the theory behind neural networks and reproducing kernel Hilbert spaces (RKHS) regression to contextualize 3 claimed benefits of DL over linear models: incorporation of diverse data types, avoidance of feature engineering, and universal approximation behavior. We also propose a taxonomy of prediction problems so that model comparisons do not confound differences in the predictive skill of different model classes with differences in the input data. Second, we leverage a maize multi-environment trial dataset to train DL and RKHS models that implicitly capture non-linear patterns between genomic, soil, weather, and management inputs and grain yield. The results demonstrate that feature engineering using principal components of SNPs generally degrades prediction accuracy across model classes. Furthermore, DL models persistently fail to outperform RKHS models across prediction problems. Finally, we evaluate the theoretical critiques with the empirical results, confirming the theoretical arguments. Nevertheless, a small portion of the possible DL model space has been explored, leaving open the possibility of DL making significant contributions to GP problems through additional aspects not considered here. We conclude by suggesting several avenues for further theoretical and practical research, including the resolution of several disciplinary differences.

## Teaser

Neural networks are theoretically advantageous for genomic prediction because they can model non-linear genotype-phenotype relationships, yet they empirically underperform classical models. Kusmec et al. compared the theoretical properties of neural networks and classical models and rigorously benchmarked them on a large, hybrid maize dataset. The results demonstrate that neural networks have fewer unique theoretical advantages than usually claimed and rarely outperform or tie classical methods. The authors argue that the potential advantages of neural networks will be realized when their implementation considers the unique structures of genotype-phenotype maps and breeding populations.

## Introduction

Prediction of phenotypes and breeding values has been an important problem since the inception of modern plant and animal breeding. Early approaches leveraged pedigree relationships to parameterize mixed linear models (Henderson 1950) for breeding value prediction and were later extended to the use of molecular markers in genomic prediction (Meuwissen et al. 2001; VanRaden 2008). Research has also focused on the prediction of phenotypes through incorporation of environmental and management data to improve prediction accuracy in new environments. Methods to incorporate these data include linear models (Jarquín et al. 2014), machine learning (Westhues et al. 2021), crop growth models (Technow et al. 2015), and deep learning (DL) (Khaki et al. 2020). Multiple studies have carried out extensive comparisons between linear, machine learning, and DL and identified small and inconsistent advantages of machine learning and DL over linear models (Bellot et al. 2018; Montesinos-López et al. 2018; Zingaretti et al. 2020). Moreover, meta-analysis reveals that, in plants, the mixed linear model (gBLUP) remains competitive and that its generalization, reproducing kernel Hilbert spaces (RKHS) regression (Morota and Gianola 2014), routinely outperforms all other methods, including neural networks/DL (Ahmadi and Bartholomé 2022).

Catalyzed by increased data availability and algorithmic and hardware advances, DL has garnered much interest for its application to prediction problems in agriculture, including phenotypic/genomic prediction. Studies (e.g. Pérez-Enciso and Zingaretti 2019; Washburn et al. 2021; Kick et al. 2023) typically make 3 claims for the advantages of DL over other methods: First, DL can naturally incorporate diverse data modalities. Second, DL obviates the need for manual feature engineering. Third, DL is a universal function approximator and can, therefore, model complex, non-linear responses. Despite these putative advantages, studies using various DL model architectures—feed-forward networks (FFNs; Kick et al. 2023), convolutional neural networks (CNNs; Bellot, de los Campos and Pérez-Enciso 2018 ), local convolutional neural networks (LCNNs; Pook et al. 2020), transformers (Chen et al. 2024), and hybrid architectures (Wang et al. 2025)—have observed variable performance relative to gBLUP and other classical models. These results are often attributed to insufficient hyperparameter optimization and/or specific datasets used for evaluation/assessment.

gBLUP is a special case of RKHS regression (Harville 1983), which uses implicit, high-dimensional, and fixed feature spaces to model complex responses. While gBLUP is the reference method for GP in most studies, RKHS models have been used to incorporate dominance deviations (Vitezica et al. 2013), epistasis (Jiang and Reif 2015), and genotype–environment interactions (Jarquín et al. 2014) into prediction models. Although since superseded for many tasks by DL in other fields, RKHS remains competitive for phenotypic and genomic prediction (Ahmadi and Bartholomé 2022).

Comparisons between classical gBLUP (and RKHS) methods and DL are complicated by model choice and intended use. For example, the linear kernel used in gBLUP models first-order polynomial relationships between the predictors and predicts breeding values (Piepho 2009). The Gaussian kernel (GK), by contrast, captures higher-order non-additive relationships (epistasis) in addition to additive relationships between the predictors (Jiang and Reif 2015) and predicts total genetic values (Morota and Gianola 2014). However, because the two models predict different quantities, scenarios where the GK model has higher prediction accuracy indicate that epistatic effects contribute significantly to the chosen evaluation metric (typically Pearson's *r* or RMSE) but do not indicate a per se superiority of the Gaussian over the linear model. Moreover, model superiority depends on the uses to which the model will be put. When recurrent population improvement is the goal, then the GK model is not useful because, in sexually reproducing organisms, alleles—not genotypes—are inherited. Therefore, inclusion of epistatic effects could alter the ranks of selection candidates relative to those based on breeding values, which are more relevant to recurrent selection. Thus, improper comparisons and failure to consider the practical uses of models can obscure under which conditions one class of models should be preferred over another.

This study argues that the inconsistent performance of FFNs (the most common DL architecture) relative to RKHS is not a consequence of insufficient optimization or data as commonly asserted; rather, it is a consequence of the non-uniqueness of the putative advantages of DL as implemented by FFNs. First, we review the theory of FFNs and RKHS and describe similarities between FFNs and RKHS with respect to incorporation of diverse data modalities, manual feature engineering, and universal approximation properties. Second, different prediction problems from the literature are distinguished by input data and predictand to clarify when comparisons between multiple models are actually informative about the relative predictive skills of the models. Third, a hybrid maize yield dataset (Kick et al. 2023) is used to investigate empirically (i) the consequences of feature engineering of genomic data in FFNs and (ii) the relative performance of RKHS and FFNs on matched prediction problems. Finally, some recommendations for the conduct and reporting of prediction studies using DL are given and key questions for future research outlined.

## Tutorial on theory and context

In this section, we provide an introduction to the theory and context of GP using neural networks and RKHS regression. We first present a summary of the mathematical and statistical theory underlying feed-forward neural networks (FFNs) and RKHS regression. Then, we evaluate the three putative advantages of DL models over classical statistical and machine learning models in light of the theoretical exposition. Finally, we present a taxonomy of GP problems to ensure that model comparisons are fair and informative regarding differences in the predictive skill of different model classes.

### An overview of FFNs

A feed-forward neural network is composed of L layers of possibly different numbers of unidirectionally connected neurons. Feed-forward neural networks generally feature fully connected layers, where the lth fully connected layer of $n_{l}$ neurons has the form


(1) $$T^{\left(l\right)}(x^{\left(l-1\right)})=\sigma (Ax^{\left(l-1\right)}+b)\;$$

where $x^{\left(l-1\right)}\in R^{n_{l-1}}$ is the vector of outputs from the (l−1)th layer (or the inputs if l−1=0); $A\in R^{n_{l}\times n_{l-1}}$ and $b\in R^{n_{l}}$ are the weight matrix and bias vector, respectively; and σ(⋅) is an activation function applied component-wise. Then the full network is given by the function composition


(2) $$F(x)=L\circ T^{\left(L-1\right)}\circ \cdots \circ T^{\left(1\right)}(x)$$

where L denotes a linear layer in this context. For (2) to be a universal approximator (see subsection 3 under “Putative advantages of deep learning” below), it is necessary that σ(⋅) be non-polynomial (Pinkus 1999), such as the popular rectified linear unit (ReLU) or sigmoid activation functions. Note that if σ(⋅) is the identity function, then (2) reduces to a standard additive regression model with non-linear constraints on the parameters. The network's performance is evaluated by a loss function. For regression problems, as in GP, the usual loss function is the root mean square error (RMSE).

The network (2) is often overparameterized, containing many more parameters than the number of observations contained in the training data, and therefore not solvable by least squares. It is typically trained by mini-batch stochastic gradient descent using the backpropagation algorithm until a predetermined number of passes over the full training dataset (or “epochs”) is complete or the loss function does not improve for a set number of epochs.

The fully connected, feed-forward architecture is only one example of a neural network. Other architectures suited to more structured inputs, such as CNNs for gridded data and recurrent neural networks for time-series data, exist. We limit ourselves to feed-forward architectures in this study because of their wide adoption in GP.

### An overview of RKHS regression

The following development closely follows that of Morota and Gianola (2014). Suppose the following model for a phenotype


(3) y=g+ε

where y is a vector of phenotypes; g is a vector of true, unknown genetic effects with distribution $N(0,\Gamma \sigma_{g}^{2})$; and ε is a vector of residuals with distribution $N(0,I\sigma_{\varepsilon }^{2})$. All vectors are n×1, where n is the number of records. Γ is the genomic relationship matrix among records using marker genotypes only at the quantitative trait loci that affect y. A prediction model generates values of unobserved phenotypes by approximating the unknown genetic values, typically as a function of *p* marker genotypes: $g=f(x_{i})$ where $x_{i}$ is the vector of (possibly) centered and scaled marker genotypes for the *i*th record. The classical choice for approximation has been the additive, linear function $f(x_{i})=x_{i}^{\prime }\beta$ , where β is a p×1 vector of marker effects. This assumption leads to the popular genomic BLUP (gBLUP) and ridge regression BLUP (rrBLUP) models, which are mathematically equivalent (Piepho 2009; Morota and Gianola 2014).

More generally, $f(x_{i})$ is the conditional expectation, or average phenotype, of individuals with markers $x_{i}$. RKHS regression approximates this function using the residual sum of squares with the squared norm of g in a Hilbert space, H, as a penalty:


(4) $$l(g|\lambda )=\|y-g{\|}^{2}+\lambda \|g{\|}_{H}^{2}$$

where *λ* controls the strength of the penalty. A Hilbert space is a generalization of the typical Euclidean space and describes a rich class of candidate functions from which an optimal functional form for g is identified. The representer theorem (Kimeldorf and Wahba 1971) guarantees that, for a given value of *λ*, the optimal g is given by the linear function Kα even when the function $f(x_{i})$ is infinite dimensional. Here, K is an n×n kernel matrix that quantifies the similarity between records, and α is an n×1 vector of regression coefficients. It can be shown that the regression coefficients are given by


(5) $$\hat{\alpha }=(K+\lambda I{)}^{-1}y$$

and then $\hat{g}=K\hat{\alpha }$.

For tractability, it is desirable to maintain the assumptions of linearity and additivity for $f(x_{i})$ in gBLUP while allowing for the possibility of a richer space of genetic predictors. We can formulate this as searching for a mapping of the *p* marker genotypes into a Hilbert space of possibly infinite dimension called the feature space, $\phi \;:R^{p}\to H$, where *ϕ* is called the feature map and $\phi (x_{i})$ is the representation of $x_{i}$ in feature space. The kernel function is then defined as the inner product in this Hilbert space:


(6) $$K(x_{i},x_{j})={\left\langle \phi (x_{i}),\phi (x_{j})\right\rangle }_{H}$$

where ${\left\langle \cdot ,\cdot \right\rangle }_{H}$ denotes the inner product in feature space. Note that a feature map uniquely defines a kernel, but the converse is not true: A kernel is associated with infinitely many feature maps. The feature map, *ϕ*, can vary widely in its dimensionality and therefore the number of operations needed to compute it. By Mercer's Theorem (Mercer 1909), a kernel function can be found that computes the inner product in feature space implicitly, that is, without evaluating the feature map in (6), which is known as the “kernel trick.”

The formulation (5) is known as kernel ridge regression and was introduced for genomic prediction by Gianola et al. (2006). Harville (1983) was the first to note that the BLUP models used by animal breeders were a special case of RKHS. The commonly used genomic relationship matrix (VanRaden 2008) in gBLUP is an application of the linear kernel where the feature map is the identity function, $\mathrm{id}(x_{i})=x_{i}$. Many possible kernels exist, and choosing a kernel that captures the structure of the data well is a critical step in defining a RKHS model (Duvenaud 2014). Some kernels possess hyperparameters, requiring some degree of optimization. Three common kernels in genomic prediction are introduced in “Materials and methods.”

For the following discussion, three important properties of RKHS models should be kept in mind. First, while RKHS relaxes gBLUP's assumption that marker effects are additive, the regression coefficients are still linear functions of the parameters, and RKHS is, therefore, a linear model. Second, a kernel defines the similarity between two records in a Hilbert space that is induced by the choice of the kernel. Different kernels amount to different choices for which features in the data are relevant to determining how similar two records are. This imposes a strong inductive bias on the model fitting procedure. Third, while the RKHS model is a linear model, the similarity induced by the choice of kernel can encode curvilinear relationships of possibly infinite dimension.

### Putative advantages of DL

The following sections contextualize the advantages claimed for DL in phenotypic/genomic prediction problems. Attention is given to similarities between DL and classical models/RKHS.

#### DL can naturally incorporate diverse data types

DL has achieved its most notable successes in computer vision and natural language processing. One contributing factor has been DL's ability to process images and video natively using tensor representations. Other inputs (real-valued predictors, one-hot encoded predictors, time-series, etc.), however, have analogs in classical statistics and machine learning. Representation of non-image/video inputs depends on devising an effective vector representation that sufficiently preserves the information and structure of the inputs. Although typically described in terms of vectors, kernel functions can be devised for diverse inputs, provided that an inner product encoding some notion of similarity or covariance exists (de los Campos et al. 2009; Morota et al. 2013). For example, SNPs for a single, diploid individual are typically represented as a vector where the elements count the number of reference alleles at each locus (0, 1, or 2). The dot product of two such vectors defines the linear kernel used for gBLUP. Alternatively, a pedigree defines a directed acyclic graph from which the same linear kernel can be derived (de los Campos et al. 2010).

#### DL avoids the need for manual feature engineering

DL models assume that informative data features can be learned through *compositionality* or repeated affine and non-linear transformations of the input data. In fields such as computer vision, this eliminates the construction of smaller sets of human-informed features. However, genomic data have proven challenging to incorporate into DL without simplifying assumptions. Although genomic language models based on transformer-like architectures have achieved numerous successes by restricting the genomic sequence context under consideration (Consens et al. 2025), dense SNP data pose different challenges for layers used in FFNs: The dimensionality is too high for computationally feasible fully connected layers, and the data violate the translation invariance assumption of convolutional layers. Some form of dimensionality reduction is typically used, such as PCA (Washburn et al. 2021; Kick et al. 2023), aggressive pre-filtering of SNPs (Bellot et al. 2018; Wang et al. 2025), or *k*-mers (Chen et al. 2024), which are forms of feature engineering. By contrast, the kernel function implicitly defines a fixed set of features, which implies a strong inductive bias on the model space (Duvenaud 2014).

#### DL is a universal function approximator

Universal approximation theorems state the conditions under which neural networks with various architectures and non-polynomial activation functions can approximate a target function with a desired, non-zero amount of error (Cybenko 1989; Hornik et al. 1989; Leshno et al. 1993). Consequently, they are often cited as alternatives to classical linear models when the target function is complex and non-linear (e.g. Kick et al. 2023). There are, however, three subtleties to this framing.

First, universal approximation theorems are *existence* theorems: They specify conditions under which such a network exists but do not provide a constructive procedure. Thus, there is no guarantee that any combination of optimization algorithm and dataset will identify such an architecture (Goodfellow et al. 2016). Second, universal approximation theorems are *limit* theorems: A network possesses the property provided that enough hidden units are present. The theorems do not, however, guarantee that any particular (finite) size is enough. Some configurations of kernels, for example, a product of GKs, possess a similar universal approximation property (Micchelli et al. 2006), which is subject to the same constraints for optimization.

Third, “linear” is polysemic in the modeling literature: Biologically, linear usually refers to a straight-line relationship between two variables, and non-linear refers to curvilinear functions—e.g. a quadratic function. “Linear” refers here to the *shape* of the functional relationship. Statistically, linear signifies that model parameters can be estimated as linear combinations of the response (Christensen 2011). In this sense, a quadratic function is a linear function while the logistic function is not. “Linear” refers here to *parameter estimation*. Thus, linear models are quite capable of representing “non-linear” relationships.

### Which prediction problem?

An underappreciated problem in the genomic prediction literature is which quantity a model predicts. Especially in prediction problems involving multi-environment trials, attention has focused on the *structure* of the data rather than the data *modalities* and how they are used by a model. For example, typical cross-validation schemes (e.g. Li et al. 2018) define different prediction problems by whether a target variety has been tested in the target environment. This stimulated research on training population design (e.g. Isidro et al. 2015) and environmental context (Costa-Neto et al. 2022) because prediction accuracy (or generalization) depends on similarity between the training and test sets.

Different models leverage this similarity to different degrees depending on the available data modalities and how they are used by the models. For instance, genetic similarity is measured using SNP genotypes. These SNPs can be used in different ways: e.g. additive coding predicts breeding values while dominance coding predicts dominance deviations. However, because quantitative genetics theory predicts that (generally) only the additive genetic variance contributes non-transiently to selection responses, the more complex joint model is only useful insofar as dominance deviations have non-zero contributions to the chosen evaluation metric and quantities other than breeding values are of interest.

**Fig. 1.**
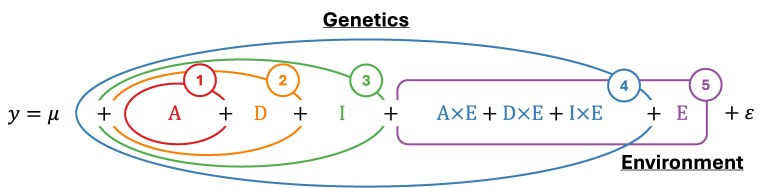
Taxonomy of prediction problems. The phenotype (y) is decomposed into a population mean (*μ*); additive (A), dominance (D), and epistatic (I) genetic effects; gene-environment interactions (A×E, D×E, and I×E); and environmental effects (E). Numbers correspond to the numbering of the prediction problems in the main text. Nested/overlapping shapes denote the hierarchical relationships between prediction problems. For full details, see “Which prediction problem?” in “Tutorial on Theory and Context.”

On the basis of quantitative genetics theory, therefore, we can identify five common prediction problems in the literature (Fig. 1). Non-transient genetic gain depends on accurate selection of individuals with high breeding values (additive genetic effects; problem 1) as the additive genetic variance is a key component of the breeder's equation (Walsh and Lynch 2018). Inclusion of dominance deviations (Vitezica et al. 2013; Costa-Neto et al. 2021) in the model is no longer prediction of breeding values but not yet total genetic values (problem 2). Inclusion of epistasis (Jiang and Reif 2015) leads to prediction of total genetic values (problem 3). Many options also exist to incorporate environmental factors or QTL-environment interactions into models (Jarquín et al. 2014; Lopez-Cruz et al. 2015; Costa-Neto et al. 2021; Washburn et al. 2021), leading to prediction of phenotypes (problem 4). Finally, use of environmental factors without genetic data predicts mean phenotypes in different environments, suitable for site-selection problems (problem 5).

Problems 1 to 4 represent a hierarchy of increasingly complex models (Fig. 1) that account for an increasing number of possible sources of phenotypic variation. If these make non-zero contributions to the chosen evaluation metric, we expect prediction accuracy to increase. Thus, comparisons between models for different problems are informative about relevant sources of variation with respect to a particular metric (see below). However, it does not follow that the top-performing model from this comparison is the “best” model. For example, only problem 1 predicts breeding values, which are the source of non-transient genetic gain. A model of type 3 might have higher prediction accuracy but would not be appropriate for the purposes of recurrent selection if the inclusion of dominance deviations and epistasis ranked selection candidates differently than breeding values did. Therefore, comparisons between different models *within the same prediction problem* are most meaningful for identifying the top-performing model for a given use.

As alluded to in the preceding discussion, the choice of evaluation metric affects the ranking of models. GP and DL models minimize similar criteria, the penalized residual sum of squares and RMSE, respectively, which are monotonically related up to a constant. However, GP models are typically assessed using Pearson's correlation coefficient, while DL models for regression tasks are tuned and assessed using RMSE. The consequences of the differences between these two metrics on model comparisons are underappreciated in the literature. Pearson's correlation coefficient assesses the strength of a first-order relationship between the observed and predicted values. If this relationship is sufficiently strong, more complex models may not substantially improve this metric. It is important to note that, for Pearson's correlation coefficient, *any sufficiently strong first-order relationship* produces coefficients close to unity regardless of the amount of bias in the predicted values. RMSE, by contrast, explicitly quantifies the deviation of the predicted values from the observed values. It can be decomposed into three orthogonal components, only one of which measures errors due to lack of correlation (Gauch et al. 2003). Thus, there may be improvements to RMSE as additional model components more accurately predict the observed *values* rather than their relative ranks, leading to re-ranking of models.

## Materials and methods

**Fig. 2.**
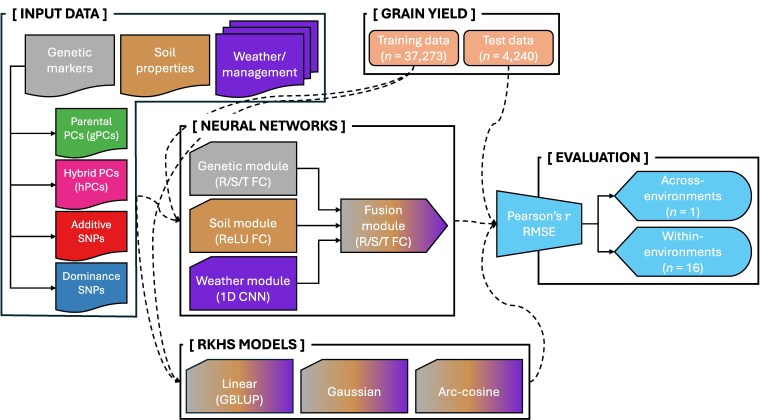
Overview of data, models, and evaluation. Input data were generated by the Genomes to Fields consortium (McFarland et al. 2020; Lima et al. 2023a, 2023b) and processed/curated by Kick et al. (2023) and Lopez-Cruz et al. (2023). Grain yield records were used to train RKHS models and neural networks. Model performance was assessed by Pearson's *r* and RMSE evaluated across-environments (main text, Figs. 3 to 8) and within-environments (Supplementary Figs. 3 to 12). Each model was fit separately to each input data type and to all 3 data types simultaneously. See “Methods” for full details. “R/S/T” indicates the use of the rectified linear unit (R; ReLU), sigmoid (S), or hyperbolic tangent (T; tanh) activation functions. FC, fully connected; 1D CNN, 1-dimensional convolutional neural network.

An overview of the data, models, and comparisons made in this study is presented in Fig. 2.

### Data overview

For a full overview of the dataset and its processing, see Kick et al. (2023). Processed data were downloaded from 10.5281/zenodo.6916775 (Kick 2022). Files beginning with “ForR” were used as input except for soil variables (see below).

Grain yield (bu/ac) was used as the response variable for all models. These values were centered and standardized by the mean and standard deviation of the training set (see below) prior to model fitting.

Genomic data were collected from two sources. First, genomic principal components (gPCs) from Kick et al. (2023) were used. gPCs were derived from genotyping-by-sequencing SNPs on the inbred parents (McFarland et al. 2020) followed by principal components analysis (PCA). One thousand and seven hundred twenty-five PCs explained >99% of the variance in the inbred genotypes. Kick et al. (2023) constructed hybrid gPCs by averaging the gPCs of the two parents, which is equivalent to first averaging the parental genotypes and then projecting them into the space spanned by the eigenvectors. Second, Lopez-Cruz et al. (2023) curated 98,206 SNPs on 3,024/3,101 G2F hybrids with minor allele frequency > 3% and LD filtering in 1 Mbp blocks Lopez-Cruz et al. (2023). These SNPs were used to fit linear, Gaussian, and arc-cosine kernel models. To compare the effects of using gPCs instead of SNPs on the performance of neural networks, a reduced set of 1,725 SNPs was constructed. Mixed model GWAS (Yu et al. 2006) as implemented by the R package “rrBLUP” (Endelman 2011) was performed in each training environment (see “Training, validation, and test sets”), including a kinship matrix and PCs of the kinship matrix selected by forward stepwise regression with BIC. *P*-values were combined using Fisher's method, and the 1,725 SNPs with the smallest combined *P*-value were used as predictors of matching input dimension for additional neural networks.

Twenty-one soil variables were included as covariates: soil pH measured using a 1:1 mixture of soil and distilled water, soil pH measured using the Woodruff method, soluble salt concentration (mmho/cm), soil organic matter (%), available nitrates (ppm), nitrogen (lbs/ac), available potassium (ppm), available sulfate (ppm), available calcium (ppm), available magnesium (ppm), available sodium (ppm), cation exchange capacity (meq/100 g soil), hydrogen (%), potassium (%), calcium (%), magnesium (%), sodium (%), phosphorus extracted by acid fluoride (ppm), and sand/silt/clay compositions (%). For replication of previous results, the file “ForRS.csv” was used. Errors in this file (D. Kick, personal communication) were corrected in new models using values from the “tensor_ref_soil.csv” file.

Nineteen weather and management variables were included as covariates: applied nitrogen/phosphorus/potassium (lbs/ac), daily minimum/mean/maximum/dew point temperatures (°C), daily mean relative humidity (%), daily mean solar radiation (W/m^2^), daily maximum wind speed (m/s), daily mean wind direction (°), daily maximum wind gust (m/s), daily mean soil temperature (°C), daily mean soil moisture (% volumetric water content), daily mean ultraviolet radiation (μmol/m^2^/s), daily photosynthetically active radiation (μmol/m^2^/s), daily mean photoperiod (h), daily estimated water vapor partial pressure (Pa), and total water applied including irrigation and precipitation (mm). Each variable was recorded for days −75 to 204 where 0 indicates the planting date.

### Training, validation, and test sets

The same train/test split defined by Kick et al. (2023) was used for model training and evaluation. Briefly, the 41,513 observations were split into training and test sets of 37,273 and 4,240 observations, respectively, by sampling site-year combinations and down-sampling hybrids within site-year combinations so that no combination accounted for more than 265 observations. Models using SNP data had 40,114 observations (36,072/4,042 train/test) available. Of 3,101 hybrids, 1,530 were unique to the training set, 12 to the test set, and 1,559 were common to both. Of 158 site-year combinations, 142 were unique to the training set and 16 to the test set. Of the 41 experimental sites, 28 were unique to the training set and 13 were in common to both. Thus, the structure of the prediction problem tested in this study is that of tested genotypes in untested environments with location and soil constant. The different data modalities used to train models define the different prediction models described in “Which prediction problem?”.

### Models

All models were fit in R on a MacBook Pro with 24 GB RAM, 8-core M3 CPU, and 10-core integrated GPU. Due to training speed and/or memory limitations, weather DL models and some interaction kernel models were run on the Kansas State University Beocat computing cluster. Memory usage did not exceed 36 GB.

#### Kernel models

RKHS models were fit in R using the Bayesian Genotype and Genotype–Environment (BGGE) package (Granato et al. 2018). The package uses an orthogonal transformation based on the eigendecomposition of the kernel matrices to reparametrize the model for an efficient Gibbs sampler (Cuevas et al. 2014). The software was modified to accept pre-computed eigendecompositions to improve efficiency when using the same kernel in different models. All models ran for 15,000 iterations with a burn-in of 5,000 iterations and a thinning period of 5 iterations.

##### Input coding

Additive genetic effects were modeled using counts of the locus-specific reference allele where


$$a_{l}=\{\begin{array}{cc} 0-2p_{l},\; & A_{1}A_{1}\\ 1-2p_{l}, & A_{1}A_{2}\\ 2-2p_{l}, & A_{2}A_{2} \end{array}$$

when $A_{2}$ is the reference allele with frequency $p_{l}$. Dominance deviations were modeled as


$$d_{l}=\{\begin{array}{cc} -2p_{l}^{2}, & A_{1}A_{1}\\ 2p_{l}q_{l},\; & A_{1}A_{2}\\ -2q_{l}^{2}, & A_{2}A_{2} \end{array}$$

where $q_{l}=1-p_{l}$, and Hardy–Weinberg equilibrium is assumed (Vitezica et al. 2013). No standardization was applied to predictors for the marker effects. Soil variables were centered and scaled. Weather and management variables were summarized as the average value across 3-day, non-overlapping windows followed by centering and scaling for each variable-window combination.

In the following equations, $n_{g}$ indicates the number of hybrids, $n_{e}$ is the number of environments, $x_{i}$ is a generic data vector for the *i*th record, X is a generic data matrix, and K is a generic kernel matrix where subscripts indicate the type of data: $K_{G}$ = gPCs, $K_{H}$ = hPCs, $K_{S}$ = soil, $K_{W}$ = weather/management, $K_{A}$ = additive genetic effects, and $K_{D}$ = dominance deviations.

##### Linear kernel

Genomic BLUP (gBLUP) is a specific instance of the linear kernel (LK), where the similarity between the *i*th and $i^{\prime }$th record is given by the inner product:


$$k_{ii^{\prime }}=\left\langle x_{i},x_{i^{\prime }}\right\rangle =\overset{p}{\underset{\;j=1}{\sum }}\;x_{ij}x_{i^{\prime }j}$$

In matrix form, the LK is computed and normalized by


$$K=\frac{{XX}^{\prime }}{\mathrm{tr}({XX}^{\prime })/n}$$

where ′ indicates matrix transposition and tr(⋅) is the trace operator (sum of the diagonal elements).

##### Gaussian kernel

The GK models similarity non-linearly using an exponential transformation of the squared Euclidean distances between records. Let $d_{ii^{\prime }}^{2}=\|x_{i}-x_{i^{\prime }}{\|}^{2}=\sum_{\;j=1}^{p}{\left(x_{ij}-x_{i^{\prime }j}\right)}^{2}$ be the squared Euclidean distance between the *i*th and $i^{\prime }$th records and *q* the median of the distances between all pairs of records. Then the GK similarity is given by


$$k_{ii^{\prime }}=\exp\;\left(-h\frac{d_{ii^{\prime }}^{2}}{q}\right)$$

where *h* is a bandwidth parameter controlling the rate of exponential decay in the pairwise similarities. The bandwidth parameter was optimized for each single-data type kernel using a maximum marginal likelihood procedure (Pérez-Elizalde et al. 2015).

##### Arc-cosine kernel

The arc-cosine kernel (AK) mimics the behavior of an infinite-width neural network with a single hidden layer (Neal 1996; Cho and Saul 2009). A recursive relationship enables the construction of a kernel that mimics the behavior of a neural network with *l* layers. The first layer uses the angle between the data vectors for the *i*th and $i^{\prime }$th records in the input space:


$$\theta_{ii^{\prime }}^{\left(0\right)}=\cos^{-1}\;\left(\frac{\langle x_{i},x_{i^{\prime }}\rangle }{\left\|x_{i}\|\|x_{i^{\prime }}\right\|}\right)$$

where the superscript indicates the *l*th layer and ‖⋅‖ the $L_{2}$ norm. Then the AK similarity for l=1 is given by


$$k_{ii^{\prime }}^{\left(1\right)}=\frac{1}{\pi }\|x_{i}\|\|x_{i^{\prime }}\|J(\theta_{ii^{\prime }}^{\left(0\right)})$$

$$J(\theta_{ii^{\prime }}^{\left(0\right)})=\sin\;(\theta_{ii^{\prime }}^{\left(0\right)})+(\pi -\theta_{ii^{\prime }}^{\left(0\right)})\cos\;(\theta_{ii^{\prime }}^{\left(0\right)})$$

Additional layers are computed recursively such that for the (l+1)th layer the similarity is given by


$$\theta_{ii^{\prime }}^{\left(l\right)}=\cos^{-1}\;\left(\frac{k_{ii^{\prime }}^{\left(l\right)}}{\sqrt{k_{ii}^{\left(l\right)}k_{i^{\prime }i^{\prime }}^{\left(l\right)}}}\right)$$

$$k_{ii^{\prime }}^{\left(l+1\right)}=\frac{1}{\pi }\sqrt{k_{ii}^{\left(l\right)}k_{i^{\prime }i^{\prime }}^{\left(l\right)}}J(\theta_{ii^{\prime }}^{\left(l\right)})$$

The number of layers was optimized using a maximum marginal likelihood procedure (Cuevas et al. 2019).

##### Hyperparameter optimization

Kernel hyperparameters were optimized using maximum marginal likelihood methods (Pérez-Elizalde et al. 2015; Cuevas et al. 2019). These methods are based on the eigendecomposition of K and become computationally expensive when K is large. To improve the computational efficiency, R functions for each procedure (Costa-Neto et al. 2021) were modified to use RSpectra (Qiu and Mei 2024) for eigendecompositions of single-data K, tensorEVD (Lopez-Cruz et al. 2024) for approximate eigendecompositions of interaction kernels, and optimParallel (Gerber and Furrer 2019) for parallelized evaluation of the GK objective function. Use of an approximate eigen basis was tested against a smaller dataset (Costa-Neto et al. 2021) and selected similar hyperparameters to an exact calculation (data not shown).

##### Weather kernel engineering

The weather and management data consisted of 19 variables summarized as the mean across 94 3-day, non-overlapping windows. Kernels were optimized separately for each variable and summed to produce a final kernel (Kick et al. 2023). For the GK and AK, a weighted average was used where the inverses of the hyperparameters were used as weights for the individual variable kernels. For the LK, there are no weights, and each environmental covariate is implicitly treated as equally important. For the GK, this procedure increased the contribution of environmental covariates with small bandwidth parameters, indicating strong, global similarity, to the composite kernel. For the AK, environmental covariates with a smaller optimum number of iterations had larger contributions to the composite kernel.

##### Kernel expansion and eigendecomposition

Kernels were calculated and optimized using unique data records for hybrids and environments. Expansion to the similarity structure of the full dataset used the Hadamard product (∘):


$$K_{AW}=(Z_{A}K_{A}{Z^{\prime }}_{A})\circ (Z_{W}K_{W}{Z^{\prime }}_{W})$$

where Z is the design matrix for the indicated data type, and data types are chosen as examples. For kernel expansion of a single-data type, one kernel matrix was replaced by an identity matrix of appropriate dimension ($I_{n_{g}}$ or $I_{n_{e}}$). An exact eigendecomposition of the expanded kernel matrix was calculated using RSpectra (Qiu and Mei 2024) with truncation of eigenvalues $<1\times 10^{-10}$ or at 7,500 eigenvalues, which captured >90% of the variance for all kernels.

##### Statistical model

Single- and multi-kernel models of the following form were fit to the training set:


$$y=1\mu +\underset{k\in K}{\sum }\;u_{k}+\varepsilon$$

where y is the response vector, ***1*** is a vector of ones, *μ* is the population mean, $u_{k}$ is the vector of random effects for the *k*th kernel with distribution $N(0,K_{k}\sigma_{k}^{2})$, and $\varepsilon ∼N(0,\sigma_{\varepsilon }^{2})$ is the vector of residuals. *k* indexes a set of kernels, K, where each element of the set has variance-covariance matrix $K_{k}$ constructed using the Hadamard product of its constituent data type. The combinations of kernels modeled are given in Table 1.

**Table 1.** Optimized kernel hyperparameters for data types and individual weather/management variables.

Data type	Variable	Gaussian kernel (h)	Arc-cosine kernel (*l*)
Genomic	Genomic PCs	4.050	6
	Hybrid PCs	0.333	7
	Additive genetic effects	0.489	5
	Dominance deviations	0.978	5
Soil	—	6.000	4
Weather/management	Nitrogen (lbs/ac)	0.050	9
	Phosphorus (lbs/ac)	5.960	18
	Potassium (lbs/ac)	6.000	34
	Min. temp. (°C)	0.050	1
	Mean temp. (°C)	0.091	1
	Max. temp. (°C)	0.127	2
	Dew point temp. (°C)	4.331	1
	Relative humidity (%)	5.820	1
	Solar radiation (W/m^2^)	6.000	3
	Max. wind speed (m/s)	6.000	1
	Mean wind direction (°)	6.000	1
	Max. wind gust (m/s)	6.000	2
	Mean soil temp. (°C)	4.887	1
	Mean soil moisture (%VWC)	6.000	1
	Mean UV radiation (μmol/m^2^/s)	2.835	1
	PAR (μmol/m^2^/s)	2.430	2
	Mean photoperiod (h)	0.238	3
	Water vapor partial pressure (Pa)	3.269	1
	Total water (mm)	6.000	1

Values for *h* are rounded to the third decimal place for convenience.

Min., minimum; Max., maximum; PAR, photosynthetically active radiation; Temp., temperature; UV, ultraviolet.

#### DL models

DL models were constructed in R using the keras3 package (Kalinowski et al. 2024) with a Tensorflow backend (Abadi et al. 2016) and the Adam optimizer (Kingma and Ba 2014 Dec 22). Hyperparameters were optimized using Bayesian optimization in the kerastuneR package (Abdullayev 2024). The hyperparameter search spaces are given in Table 2.

**Table 2.** Hyperparameter search spaces for deep learning models.

Category	Model	Hyperparameter	Range
Architecture	Genomic	Layers	1 to 7
		Units per layer	4 to 256
		Dropout rate	0 to 0.3
	Soil	Layers	1 to 7
		Units per layer	4 to 64
		Dropout rate	0 to 0.3
	Weather/management	Pooling type (1D)	Maximum, average
		Convolution blocks	1 to 7
		Convolution layers per block	1 to 4
		Filters per layer	4 to 512
	Fusion	Layers	1 to 7
		Units per layer	4 to 64
		Dropout rate	0 to 0.3
Training	Optimizer	Learning rate	0.1, 0.01, 0.001, 0.0001
		$\beta_{1}$	0.9 to 0.9999
		$\beta_{2}$	0.9 to 0.9999
	Other	Batch size	32 to 256, step = 16
		Epochs	1 to 1,000

Optimized hyperparameters are given in Table 3 (architecture) and Table 4 (training).

Soil data were fit by a feed-forward, fully connected network using ReLU activation functions. Weather data were fit by a 1D convolutional network using ReLU activation functions. These 2 network architectures were unchanged throughout to isolate the effects of different genomic data types and activation functions on prediction accuracy. Genomic data types were fit by feed-forward, fully connected networks using ReLU, sigmoid, and tanh activation functions. Fusion modules were feed-forward, fully connected networks using the activation function corresponding to that of their genomic sub-module.

The training data were split into 10 training/validation sets by sampling 16 site-year combinations from the 142 training combinations. A custom tuning class randomly selected one training/validation pair for each hyperparameter set to avoid overfitting to a single validation set. A maximum of 40 hyperparameter sets were explored for each DL model with training up to 500 epochs and an early stopping patience of 7 epochs. The 4 best performing hyperparameter sets for each model were then trained for 500 epochs on each of the 10 training/validation sets. For each hyperparameter set, the mean and standard deviation of the validation loss were calculated across training/validation pairs for each epoch. Losses were binned into 10 bins of width 50 epochs each with bin loss defined as the average of the summed validation mean and standard deviation loss for each epoch. The hyperparameter set that minimized this binned loss over the most bins was chosen as the optimal hyperparameter set. The optimum number of epochs was chosen as the earliest epoch at which the 20-epoch rolling mean validation loss summed across the 10 training/validation pairs was minimized. Optimized architectures and optimizer parameters are given in Tables 3 and 4, respectively.

**Table 3.** Optimized architecture hyperparameters for deep learning models.

				Specific layer						
Model	Activation function	Genomic data	Hyperparameter	1	2	3	4	5	6	7
Genomic	ReLU	gPCs	Units	192	130	156	42	55	189	—
			Dropout rate	0.153	0.137	0.275	0.105	0.118	0.078	—
		hPCs	Units	217	—	—	—	—	—	—
			Dropout rate	0.159	—	—	—	—	—	—
		SNPs	Units	31	—	—	—	—	—	—
			Dropout rate	0.097	—	—	—	—	—	—
	Sigmoid	gPCs	Units	81	64	—	—	—	—	—
			Dropout rate	0.053	0.188	—	—	—	—	—
		hPCs	Units	96	212	—	—	—	—	—
			Dropout rate	0.014	0.201	—	—	—	—	—
		SNPs	Units	201	98	—	—	—	—	—
			Dropout rate	0.130	0.202	—	—	—	—	—
	Tanh	gPCs	Units	197	—	—	—	—	—	—
			Dropout rate	0.176	—	—	—	—	—	—
		hPCs	Units	118	99	90	103	70	—	—
			Dropout rate	0.146	0.212	0.078	0.145	0.148	—	—
		SNPs	Units	244	54	—	—	—	—	—
			Dropout rate	0.029	0.081	—	—	—	—	—
Soil	ReLU	gPCs	Units	45	35	56	9	4	4	4
			Dropout rate	0.278	0.232	0.207	0.064	0	0	0
		hPCs/SNPs	Units	55	47	51	54	—	—	—
			Dropout rate	0.235	0.222	0.237	0.258	—	—	—
Weather/management	ReLU	gPCs	Pooling type (1D)	Maximum						
			Convolution blocks	2						
			Layers per block	1						
			Filters per layer	303	4	—	—	—	—	—
		hPCs/SNPs	Pooling type (1D)	Maximum						
			Convolution blocks	1						
			Layers per block	2						
			Filters per layer	463	378	—	—	—	—	—
Fusion	ReLU	gPCs	Units	5	36	9	34	32	—	—
			Dropout rate	0.074	0.115	0.160	0.128	0.282	—	—
		hPCs	Units	48	58	63	40	18	24	19
			Dropout rate	0.082	0.214	0.195	0.022	0.080	0.286	0.193
		SNPs	Units	39	13	53	—	—	—	—
			Dropout rate	0.165	0.294	0.232	—	—	—	—
	Sigmoid	gPCs	Units	41	17	—	—	—	—	—
			Dropout rate	0.089	0.200	—	—	—	—	—
		hPCs	Units	28	42	43	21	—	—	—
			Dropout rate	0.070	0.252	0.274	0.165	—	—	—
		SNPs	Units	43	42	31	25	50	—	—
			Dropout rate	0.145	0.082	0.052	0.197	0.033	—	—
	Tanh	gPCs	Units	55	—	—	—	—	—	—
			Dropout rate	0.188	—	—	—	—	—	—
		hPCs	Units	55	31	19	—	—	—	—
			Dropout rate	0.169	0.049	0.265	—	—	—	—
		SNPs	Units	45	32	—	—	—	—	—
			Dropout rate	0.150	0.265	—	—	—	—	—

Hyperparameter search spaces are given in Table 2. Dashes (“—”) indicate a layer not chosen by hyperparameter optimization. Hyperparameters constrained to be identical across layers are centered. Dropout rates are rounded to the third decimal place for convenience.

**Table 4.** Optimized training hyperparameters for deep learning models.

			O			ptimizerOther	
Model	Activation function	Genomic data	Learning rate	β _1_	β _2_	Batch size	Epochs
Genomic	ReLU	gPCs	0.0001	0.986	0.964	48	493
		hPCs	0.0001	0.903	0.995	176	51
		SNPs	0.0001	0.992	0.994	112	36
	Sigmoid	gPCs	0.01	0.901	0.921	64	253
		hPCs	0.0001	0.957	0.933	32	46
		SNPs	0.01	0.959	0.957	112	267
	Tanh	gPCs	0.01	0.967	0.966	208	297
		hPCs	0.01	0.937	0.963	192	22
		SNPs	0.001	0.937	0.916	160	23
Soil	ReLU	gPCs	0.0001	0.956	0.995	240	141
		hPCs/SNPs	0.0001	0.998	0.947	256	404
Weather/management	ReLU	gPCs	0.001	0.955	0.924	48	124
		hPCs/SNPs	0.001	0.916	0.999	256	113
Fusion	ReLU	gPCs	0.001	0.948	0.986	112	492
		hPCs	0.1	0.987	0.931	176	409
		SNPs	0.01	0.921	0.905	48	59
	Sigmoid	gPCs	0.01	0.948	0.984	256	86
		hPCs	0.01	0.910	0.971	32	25
		SNPs	0.01	0.971	0.968	128	384
	Tanh	gPCs	0.01	0.930	0.922	160	30
		hPCs	0.001	0.905	0.991	160	60
		SNPs	0.001	0.979	0.933	80	20

Hyperparameter search spaces are given in Table 2. Values for $\beta_{1}$ and $\beta_{2}$ are rounded to the third decimal place for convenience.

Outputs of the final layers of the genomic, soil, and weather networks were concatenated as inputs to the fusion network. To account for sampling variability among the 10 initializations of each network (see “Model evaluation”), the interaction network was optimized by randomly selecting a training/validation pair and one random seed from each of the single-data networks. Similarly, 10 concatenated datasets were assembled by randomly selecting the outputs from one of the 10 single-data networks for each data modality and used to train 10 replicates of the optimized interaction network. Note that this differs from the procedure of Kick et al. (2023), which selected the output of a single random initialization of the single-data networks to serve as the training data for 10 random initializations of the fusion network.

### Model evaluation

Each model-data combination was run 10 times using different random seeds for the Gibbs sampler (RKHS) or initial weights (DL) to assess the consistency of performance. Predicted yields for the test set using different subsets of the data types were used to calculate RMSE and Pearson's correlation (*r*) within- and across-environments. Significant differences in performance were assessed using Dunnett's test implemented in the R package “multcomp” (Hothorn et al. 2008). Better models were those with performance metrics greater (*r*) or less (RMSE) than a suitable control selected according to each prediction problem.

## Results

The performance of the fitted FFNs and RKHS models are presented in three main subsections. First, we correct two errors in the results of Kick et al. (2023). Second, we extend the corrected models to further investigate the consequences of feature engineering by PCA for SNP predictors on the relative performance of FFNs and RKHS. Third, using the problem taxonomy in “Theory,” we present comparisons of the two model classes, investigating their relative performance when the complexity of the prediction problem varies.

### Correcting past results

To better connect our work with past results, we replicated the gBLUP and FFN (DNN-CO) models from Kick et al. (2023). This effort revealed two errors in that study. First, inspection of source code revealed that while the text indicated the use of the GK in the gBLUP models, a linear kernel (traditional gBLUP; LK) was fitted. This was confirmed by comparison of our replicated results with their Table 5 (Supplementary Fig. 1). Second, inspection of the source data revealed an error in the processing of the soil data (D. Kick, personal communication). Correction greatly improved the performance of the soil only models (see “Problem 5” below). Finally, while hyperparameter optimization chose different architectures, FFN results remained comparable (Supplementary Fig. 2) with two exceptions. First, correction of the soil data improved model performance. Second, the variability of the gPC-soil-weather (GSW) model increased due to differences in the training procedure (see “Discussion”).

### Feature engineering of genomic data

Kick et al. (2023) transformed inbred parent SNPs into principal components and constructed hybrid “genotypes” as the averages of the corresponding first 1,725 parental PCs (>99% variance; gPCs). To examine the impact of this transformation on prediction accuracy, 3 additional sets of genomic predictors were constructed using 98,206 SNPs (Lopez-Cruz et al. 2023) and compared to our corrected models. First, kernel regression models using all 98,206 SNPs were fitted to test the use of untransformed genomic data. Second, the top 1,017 PCs of the hybrid SNP matrix (>99% variance; hPCs) were used to compare the estimation of PCs in the parental vs hybrid populations. Third, 1,725 GWAS-significant SNPs in the training data were selected to enable the training of FFNs on untransformed genomic data.

**Fig. 3.**
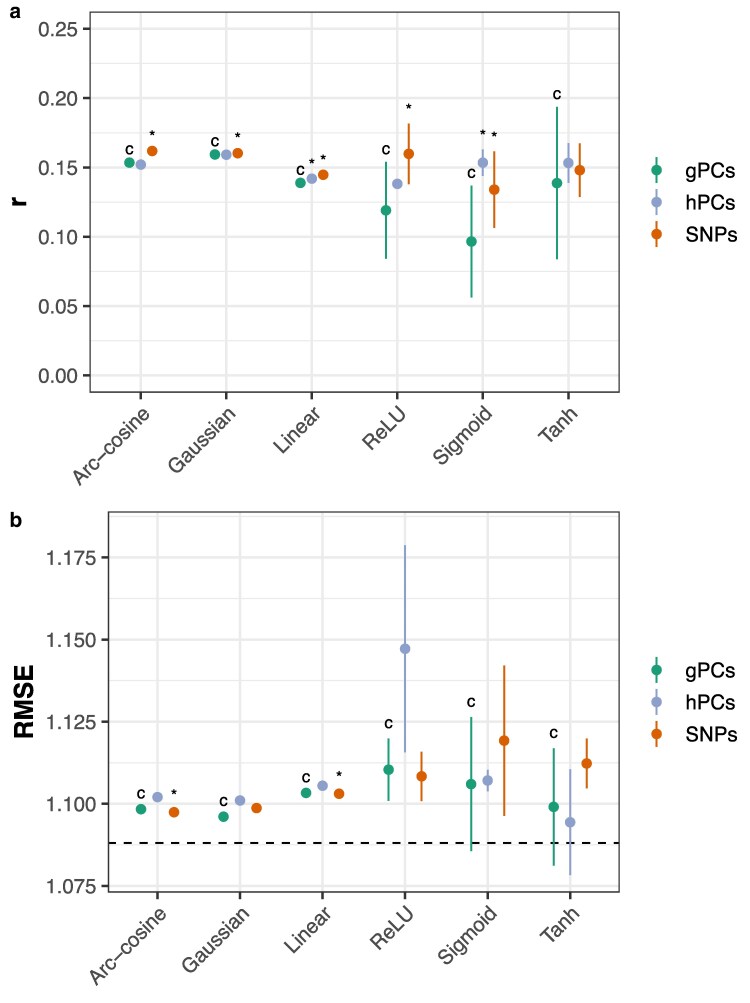
Prediction accuracy using genomic PCs (gPCs), hybrid PCs (hPCs), or SNPs as inputs. Accuracy is measured by a) Pearson's *r* or b) RMSE on an independent test set. Points indicate the mean of 10 replicates with different random seeds; vertical lines, ±3 standard errors of the mean. The horizontal line indicates the RMSE for an intercept-only model. “*” indicates p≤0.05 for Dunnett's test against the control (“c”) condition that Pearson's *r* is larger or RMSE is smaller for different genomic data types within each model class.

SNP-based FFNs (ReLU, sigmoid, and tanh) and RKHS (linear, Gaussian, and arc-cosine) models consistently outperformed gPC and hPC models across-environments (Fig. 3; Dunnett's test, *p* < 0.05). hPCs marginally improved prediction accuracy relative to gPCs for the linear kernel (gBLUP) model and sigmoid FFNs (Fig. 3). Notably, the ReLU network trained on 1,725 SNPs achieved comparable accuracy to the arc-cosine and GK models using all 98,206 SNPs and outperformed the linear kernel model (two-sample *t*-test, *p* = 0.034). Tanh networks were insensitive to transformations of the input data and uniformly matched the performance of the top-performing arc-cosine, SNP-based RKHS model (two-sample *t*-tests, *p* > 0.05). When assessed within-environments, hPCs were typically the best predictors for sigmoid FFNs, and SNPs were the best predictors for tanh FFNs (Supplementary Figs. 3 and 4). Results for other models were similar to the across-environments comparison. These results suggest that principal components—computed on either population—are not adequate genomic predictors in this population.

Using gPCs as predictors assumes that the directions of greatest variance in the parental genetic space are aligned with phenotypic variation. By constructing hybrid PCs as the average of the corresponding gPCs, Kick et al. (2023) further assumed that the directions of greatest variance are the same in both populations. This is not a well-founded assumption. To see this, let $X\in \{0,1,2{\}}^{n\times p}$ be the matrix of *p* marker calls for *n* inbred parents and $M\in \{0,1{\}}^{m\times n}$ a binary matrix linking the *m* hybrids to their parent inbreds. Then the hybrid markers are given by $\frac{1}{2}MX$. hPCs are constructed from the eigendecomposition of the p×p covariance matrix between SNPs (i.e. patterns of linkage disequilibrium):


$$\begin{array}{rl} S^{\mathrm{hybrid}} & =\frac{1}{4m}(MX{)}^{\prime }(MX)\\ & =\frac{1}{4m}X^{\prime }(M^{\prime }M)X \end{array}$$

The matrix $M^{\prime }M$ is symmetric and positive semi-definite with diagonal entries equal to the number of progeny each inbred parent has in the hybrid population and 1's in the off-diagonal entries for each observed pairing of inbred parents. All other entries are 0. We can re-write this contribution matrix as $M^{\prime }M=V\Lambda V^{\prime }$, where V is the n×n matrix of eigenvectors denoting the axes of variation in inbred parent contributions and Λ is the diagonal matrix of their *n* eigenvalues. We then have


$$\begin{array}{rl} S^{\mathrm{hybrid}} & =\frac{1}{4m}\left(X^{\prime }V\Lambda^{\frac{1}{2}}\right)\left(\Lambda^{\frac{1}{2}}V^{\prime }X\right)\\ & =\frac{1}{4m}{QQ}^{\prime } \end{array}$$

where $Q=X^{\prime }V\Lambda^{\frac{1}{2}}$. Collectively, the columns of $X^{\prime }$ represent the population of *n* inbred parents in *p*-dimensional genetic space. Multiplication by the scaled eigenvectors of $M^{\prime }M$ rotates and scales the parental representations by their patterns of contribution to the hybrid population. In the testcross design used for the Genomes to Fields (G2F) experiments, we expect these patterns of contribution to align most strongly with the testers and any associated population structure and to compress the variation into a smaller genetic subspace (compare Fig. 1 of Lopez-Cruz et al. 2023). Consequently, ∼700 fewer PCs are required to capture 99% of the variation in the hybrid population compared with the inbred parents.

### Problem-specific comparison of model performance

Plant breeders face prediction problems of varying complexity from the prediction of breeding values in population improvement to the prediction of phenotypes across multi-environment trials. Different data types (e.g. genomic vs weather data) are required for different prediction problems (Fig. 1). It is, therefore, important to benchmark models on the same prediction tasks. This subsection presents comparisons of model performance using the five problems outlined in “Theory.”

#### Problem 1: FFNs do not reliably outperform gBLUP

**Fig. 4.**
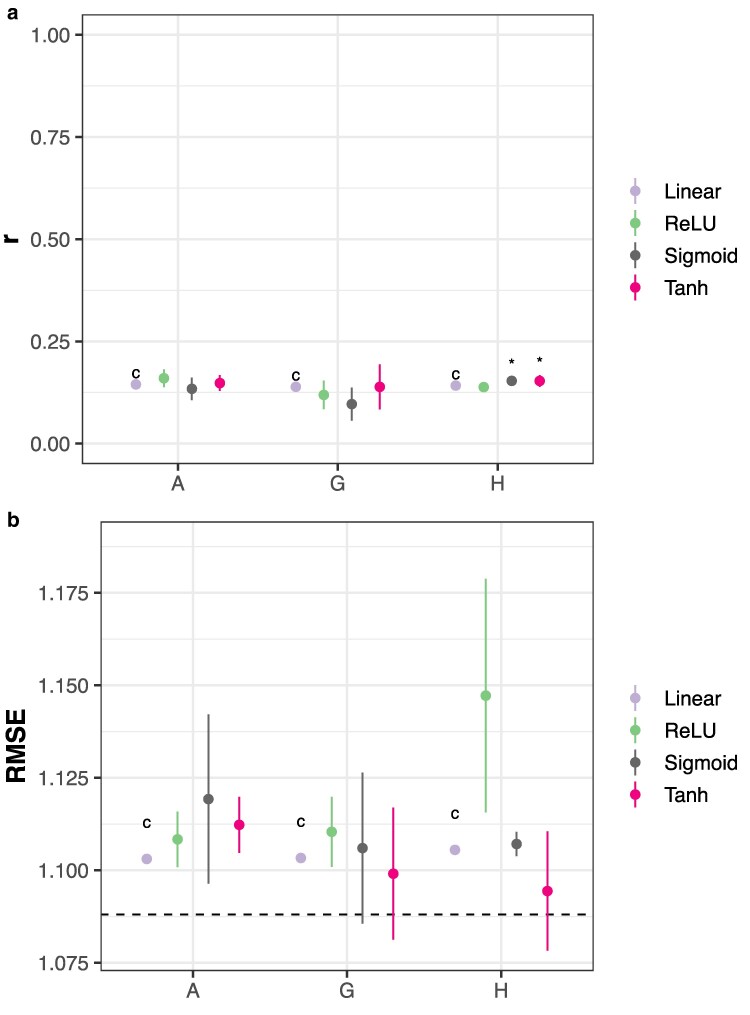
Breeding value prediction accuracy using a linear kernel (RKHS) or neural networks. Accuracy is measured by a) Pearson's *r* or b) RMSE on an independent test set. Points indicate the mean of 10 replicates with different random seeds; vertical lines, ±3 standard errors of the mean. The horizontal lines indicate RMSE for an intercept-only model. The horizontal line indicates the RMSE for an intercept-only model. “*” indicates p≤0.05 for Dunnett's test against the control (“c”) condition Pearson's *r* is larger or RMSE is smaller for different model classes within each genomic data type. “G” indicates the use of genomic PCs only; “H,” the use of hybrid PCs only; and “A,” the use of additive genetic effects only.

BLUP, whether using the numerator or genomic relationship matrices, has been the mainstay of breeding value prediction since its introduction. Linear kernel (gBLUP) models were compared with FFNs using 3 different activation functions and the genomic datasets discussed previously. Using genomic data only, only sigmoid and tanh FFNs marginally outperform the linear kernel using hPCs (Fig. 4; Dunnett's test, *p* < 0.05). When assessed within-environments, FFNs were outperformed by the linear kernel using additive genetic effects or hPCs in 7/16 environments when assessed by Pearson's *r* (Supplementary Fig. 5) but not RMSE (Supplementary Fig. 6). However, the environments and activation functions with higher performance were not consistent across genomic data types.

#### Problem 2: hybrid prediction is worst using gPCs

**Fig. 5.**
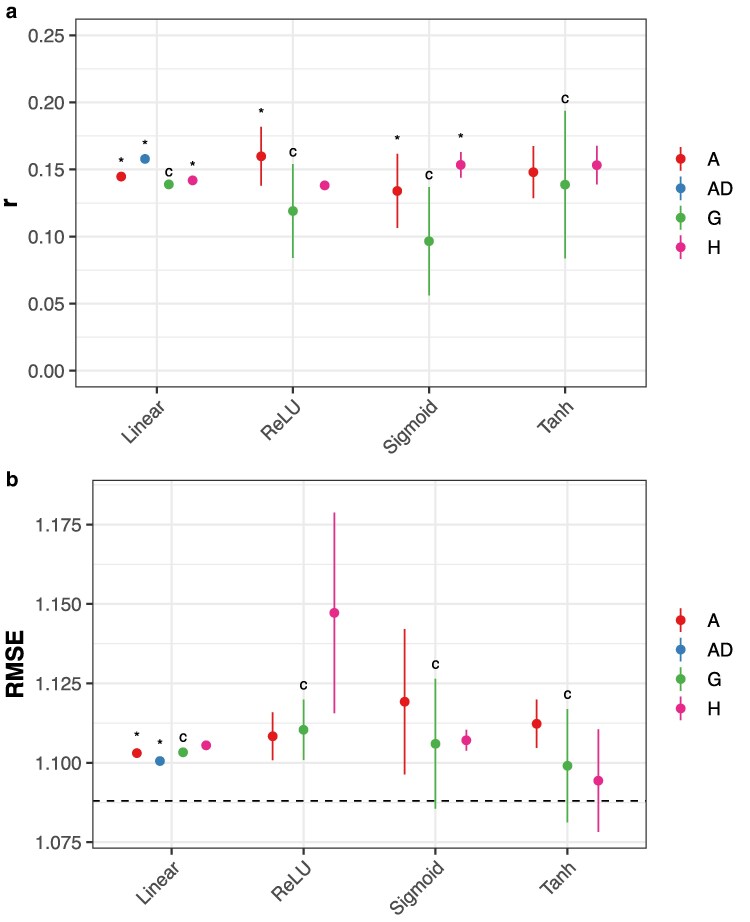
Hybrid prediction accuracy using RKHS models or neural networks. Accuracy is measured by a) Pearson's *r* or b) RMSE on an independent test set. Points indicate the mean of 10 replicates with different random seeds; vertical lines, ±3 standard errors of the mean. The horizontal line indicates the RMSE for an intercept-only model. “*” indicates p≤0.05 for Dunnett's test against the control (“c”) condition that Pearson's *r* is larger or RMSE is smaller between genomic data types within each model class. “G” indicates the use of genomic PCs; “H,” the use of hybrid PCs; “A,” the use of additive genetic effects; and “D,” the use of dominance deviations.

Because maize is grown as a hybrid crop, the G2F trials use a testcross design. Thirty-nine inbreds were used as testers for >100 hybrids each in these experiments, accounting for 40,090/41,513 (96.5%) of the observations. More generally, hybrid prediction is a challenging problem because the number of possible hybrids from *n* inbreds scales quadratically. The gBLUP (linear kernel) model and FFNs were compared using different genomic data types as in Fig. 3 with the addition of dominance deviations for the linear kernel. Dominance deviations provided a significant advantage for the linear kernel model; SNPs improved the prediction accuracy of ReLU and sigmoid FFNs; and hPCs improved the prediction accuracy of sigmoid FFNs (Fig. 5; Dunnett's test, *p* < 0.05). gPCs remained the least useful genomic predictors both across- (Fig. 5) and within-environments (Supplementary Figs. 7 and 8).

#### Problem 3: RKHS is more consistent than FFNs

**Fig. 6.**
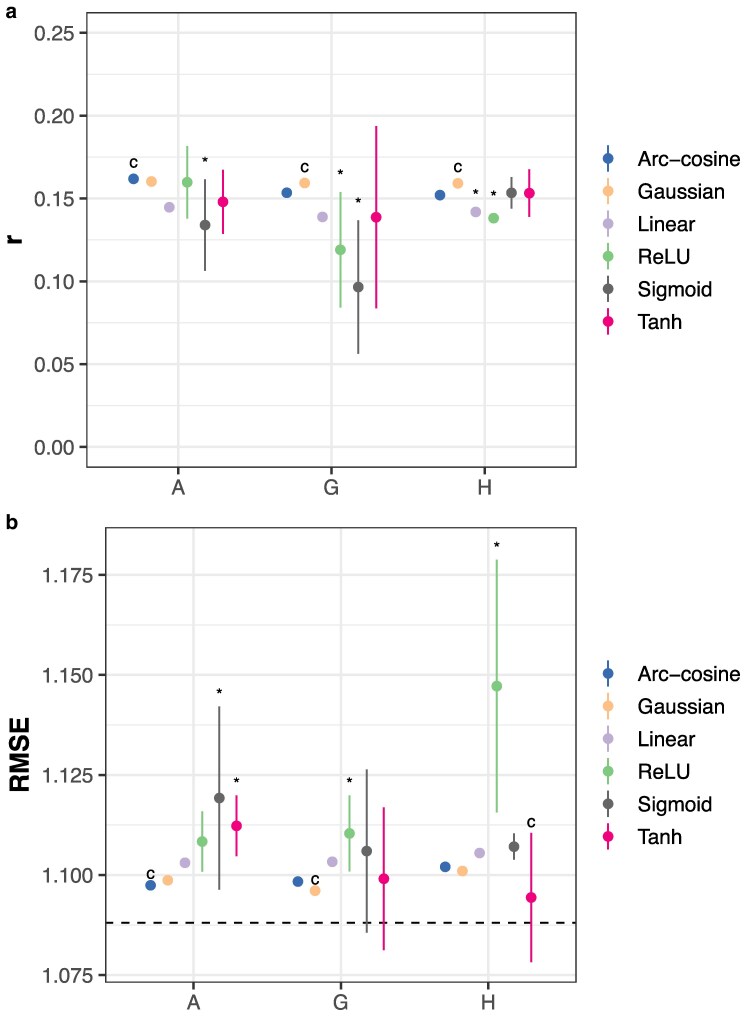
Total genetic value prediction accuracy using RKHS models or neural networks. Accuracy is measured by a) Pearson's *r* or b) RMSE on an independent test set. Points indicate the mean of 10 replicates with different random seeds; vertical lines, ±3 standard errors of the mean. The horizontal line indicates the RMSE for an intercept-only model. “*” indicates p≤0.05 for Dunnett's test against the control (“c”) condition that Pearson's *r* is smaller or RMSE is larger between model classes within each genomic data type. The control condition is chosen to be the model class with the largest average Pearson's *r* or smallest average RMSE. “G” indicates the use of genomic PCs only; “H,” the use of hybrid PCs only; and “A,” the use of additive genetic effects only.

gBLUP is a special case of RKHS using a linear kernel. Models using Gaussian and arc-cosine kernels were also fitted using different genomic data types to evaluate the impact of including epistatic effects. RKHS models using the Gaussian and arc-cosine kernels were typically the best-performing models on average with negligible variation across initializations, especially compared with FFNs (Fig. 6). FFNs exhibited substantial variation in performance, especially within-environments (Supplementary Figs. 9 and 10) where they were most likely to differ significantly from the best-performing model.

#### Problem 4: RKHS with genomic data predicts phenotypes best

**Fig. 7.**
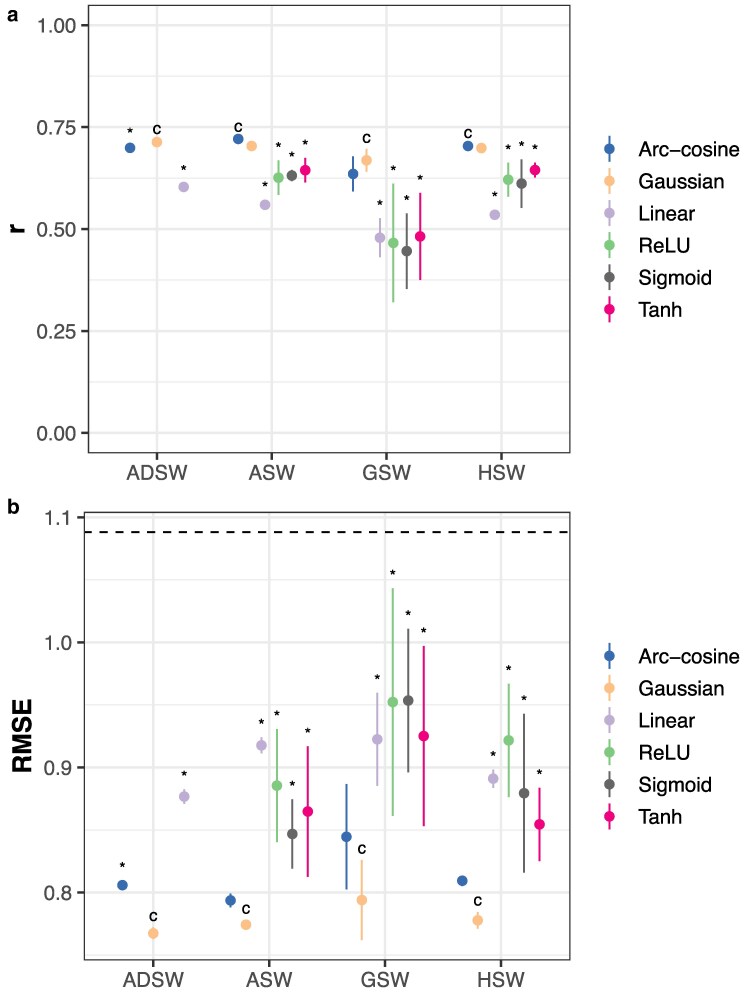
Phenotypic prediction accuracy using RKHS models or neural networks. Accuracy is measured by a) Pearson's *r* or b) RMSE on an independent test set. Points indicate the mean of 10 replicates with different random seeds; vertical lines, ±3 standard errors of the mean. The horizontal line indicates the RMSE for an intercept-only model. “*” indicates p≤0.05 for Dunnett's test against the control (“c”) condition that Pearson's *r* is smaller or RMSE is larger between model classes within genomic data types. The control condition is chosen to be the model class with the largest average Pearson's *r* or the smallest average RMSE. All models use soil (“S”) and weather (“W”) data and their 2-way interactions with genetic data. “G” indicates the use of genomic PCs; “H,” the use of hybrid PCs; “A,” the use of additive genetic effects; and “D,” the use of dominance deviations.

Models using all data modalities and their interactions were fitted to predict phenotypes. Gaussian and arc-cosine kernel models showed a clear and consistent advantage over other model classes regardless of data type when evaluated across-environments (Fig. 7; Dunnett's test, *p* < 0.05). When evaluated by RMSE, the GK model was the best performing model on average regardless of the input data (Fig. 7b). All 3 FFNs performed similarly in this analysis as a result of the strength of the soil and weather sub-models (see “Problem 5” below) but were persistently and significantly worse than the control models in all comparisons (Fig. 7). The same consistent superiority of RKHS models to FFNs was observed when evaluated within-environments, as well (Supplementary Figs. 11 and 12). In a follow-up study, Kick and Washburn (2023) constructed ensembles of the models from Kick et al. (2023). Notably, our additive/dominance GK model with soil and weather (ADSW) interactions substantially outperformed the best ensemble from this latter study when measured by RMSE (0.767 vs 0.872, respectively).

#### Problem 5: a linear kernel best predicts site-specific quality

**Fig. 8.**
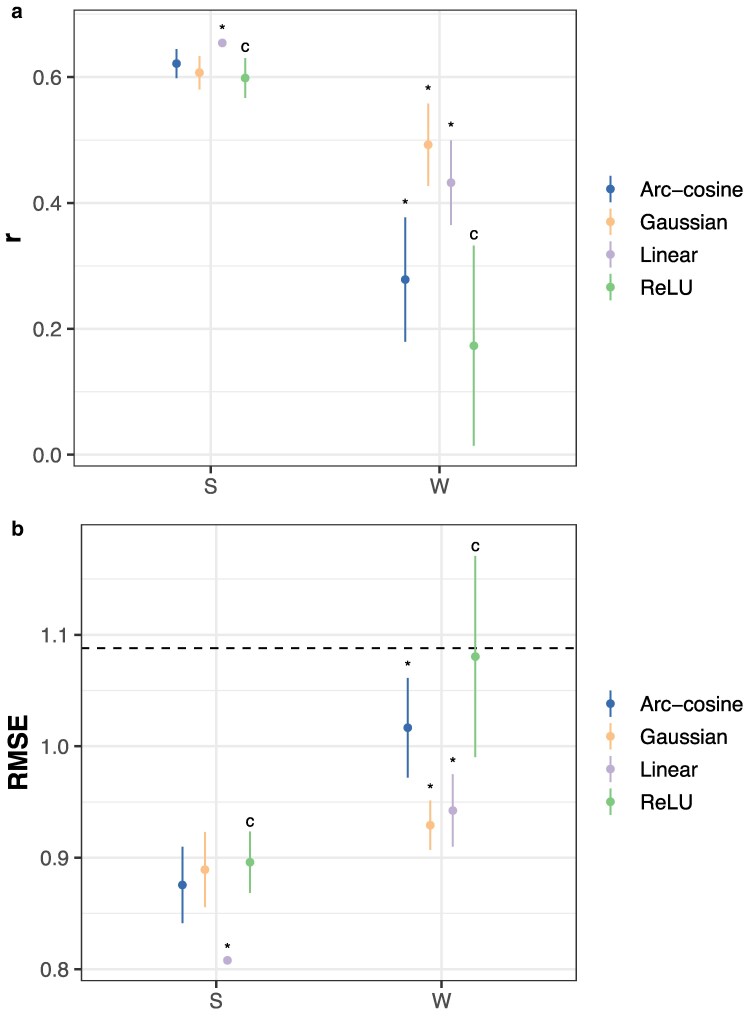
Site-mean prediction accuracy using RKHS models or a neural network. Accuracy is measured by a) Pearson's *r* or b) RMSE on an independent test set. Points indicate the mean of 10 replicates with different random seeds; vertical lines, ±3 standard errors of the mean. The horizontal line indicates the RMSE for an intercept-only model. “*” indicatesp≤0.05 for Dunnett's test against the control (“c”) condition that Pearson's *r* is larger or RMSE is smaller between model classes within data types. “S” indicates the use of soil data only. “W” indicates the use of weather data only.

Selection of informative experimental sites is an important question in the establishment and maintenance of testing networks. Here, interest mainly lies in the prediction of aggregated performance rather than the performance of individual varieties. Models using only soil or weather and management data are suitable for this task, although in the absence of genotypic data to differentiate varieties, they will predict mean performance at each site. Surprisingly, a linear kernel model consistently outperformed all other models at this task (Fig. 8; Dunnett's test, *p* < 0.05).

## Discussion

Prediction of breeding values and performance continues to be an important problem in plant and animal breeding and will continue to be so as agricultural systems strive to meet growing demands in an era of global climate change. The development of predictive models using the mixed linear model framework during the 20th century and then the development of whole-genome regression in the 21st century have been important contributors to the successes of breeding in the past 100 years. Much research has focused on leveraging new technologies for multi-modal, high-throughput data collection in increasingly complex prediction scenarios. DL has been a leading candidate for a new generation of predictive models for breeding due to three perceived advantages: natural incorporation of multiple data modalities, learned data features, and universal function approximation behavior. Empirically, however, these models perform inconsistently (Bellot et al. 2018; Montesinos-López et al. 2018; Zingaretti et al. 2020) and are typically outperformed by classical competitors, particularly RKHS (Ahmadi and Bartholomé 2022), as also demonstrated in this study.

The failure of DL to significantly outperform classical models is typically ascribed to a lack of data and/or hyperparameter optimization (Bellot et al. 2018; Montesinos-López et al. 2018; Azodi et al. 2019; Crossa et al. 2019; Pérez-Enciso and Zingaretti 2019; Zingaretti et al. 2020). This study took advantage of a large (*n* = 41,513) dataset and conducted extensive hyperparameter tuning (40 parameter sets followed by 10-fold cross-validation of the top 4 sets for each model) to train 18 feed-forward neural networks (FFNs), finding that the resulting FFNs consistently underperformed classical models. By direct comparison with RKHS, this study argues that this discrepancy stems from the non-uniqueness of DL with respect to incorporation of diverse data modalities and approximation of non-linear functions. With respect to data modalities, it is relatively trivial to incorporate environmental data into RKHS models as has been extensively demonstrated in the literature (e.g. Jarquín et al. 2014). By contrast, FFNs required extensive hyperparameter optimization for additional network modules, and the optimization of 1D CNNs for the weather data was particularly computationally costly. With respect to universal approximation behavior, the universal approximation theorems for FFNs are weaker guarantees than typically assumed (Goodfellow et al. 2016), providing the conditions under which a given network may be a universal approximator but not a constructive procedure. Furthermore, certain RKHS models are also universal approximators (Micchelli et al. 2006). RKHS models typically outperformed FFNs across the five prediction problems in this study, demonstrating the difficulty of constructing and training an FFN that is a universal approximator.

The one exception to the three potential advantages is feature engineering. Current applications of FFNs use some manual feature engineering, typically to reduce the dimensionality of high-throughput genomic data. This is a computational or representational issue and could conceivably be solved. By contrast, the choice of kernels in RKHS imposes a fixed feature space on the data and is readily scalable to large genomic datasets. This study evaluated the impact of feature engineering on genomic data using principal components, which capture patterns of linkage disequilibrium in the training population. Models using PCs derived from the parental (gPC) and hybrid (hPC) populations were consistently outperformed or matched by models using SNPs across all model classes. Generally, FFN performance suffered when using gPCs, and hPCs provided no clear benefits. Two notable findings emerged from these comparisons. First, the performance of the ReLU network using SNPs matched the SNP-based arc-cosine model (two-sample *t*-test, *p* > 0.05); however, it did not significantly outperform the ReLU network using hPCs, which exhibited much more stable performance. Second, networks using the tanh function were insensitive to transformations of the input data and exhibited comparable performance to the SNP-based arc-cosine model (two-sample *t*-tests, *p* > 0.05). These findings highlight the potential for meaningful interactions between input data and activation function that should be investigated further. Given the diversity of DL architectures, there is room for further exploration that may better leverage the structure of genomic marker data.

Overall, our empirical results confirm the theoretical predictions. We note three broad conclusions. First, the optimized FFNs rarely outperform the RKHS models for any prediction problem and set of inputs when measured by Pearson's correlation. Parity is more often achieved when performance is measured by RMSE, which is the metric that the FFNs are trained to minimize. However, Pearson's *r* is the more relevant metric for GP problems when the goal is identification of selection candidates because it is a component of the breeder's equation and governs the expected selection response. Conversely, if performance prediction is the goal, RMSE is the more relevant metric. Second, RKHS models with non-linear kernels (Gaussian and arc-cosine) consistently outperform FFNs on matched prediction problems. Indeed, FFNs struggle to outperform linear kernel (gBLUP) models in many scenarios. This suggests that the FFNs did not converge to architectures capable of modeling curvilinear relationships in this dataset. This was robust over the five different prediction problems. Third, we documented high variability in the predictions generated by the FFNs and particularly the fusion FFNs relative to RKHS models. To propagate uncertainty through hyperparameter optimization, we randomly selected different initializations of the single-data source models as inputs to the fusion models instead of fixing these inputs at a single initialization. This more accurately quantifies the stability of the predictions made by consecutively optimized FFNs and shows that they are unstable relative to those made by RKHS models.

Given the claims made in the literature and the arguments and results of this study, two questions arise with respect to the potential superiority of DL for prediction problems in breeding: How much data? How much hyperparameter optimization? While technological advances have increased the amount of data that can be gathered in a breeding program, datasets of the scale used to train successful models for other problems (notably image classification and natural language processing) are unlikely to be obtainable by individual public breeding programs. Given the typical complexity of the GP problem, the flexibility of DL models, and a small dataset, it seems highly likely that the model structure would be underdetermined to such an extent that a good approximation would be learned with low probability. This may be analogous to the observation that typical GP datasets are not large enough to induce learning in the Bayesian alphabet models (Lehermeier et al. 2013). Additionally, GP studies using DL manually define a small set of architectures and then search the corresponding hyperparameter spaces for a single best model. More resources could always be devoted to more thorough and/or larger searches (Crossa et al. 2019) but without guaranteed performance improvements. An alternative strategy would be the construction of design spaces—populations of network architectures with a high concentration of good models (Radosavovic et al. 2020). This strategy has been effective for identifying efficient, high-performing CNN architectures for image classification (Radosavovic et al. 2020).

Given the importance of the prediction problem to agriculture, continued exploration of DL and other methods is warranted; however, the empirical results of this and other studies and the theoretical considerations detailed here suggest four improvements for future research. First, classical models are firmly grounded in quantitative genetics theory with ready biological interpretation even for superficially unrelated model classes such as RKHS (Morota and Gianola 2014; Jiang and Reif 2015). By contrast, most applications of DL to GP have involved relatively straightforward translation of common architectures from other fields to the GP domain (two recent exceptions are Pook et al. 2020 and Kihlman et al. 2024). Critical assessment of the structure of GP problems and the conditions under which DL might improve over classical methods would help guide future research. For example, CNNs assume that the input data are translation invariant, which applies the same convolutional filter with fixed width to SNPs across the genome. This is manifestly unrealistic for SNPs that are distributed non-uniformly across the genome and whose effects on phenotypic variation are strongly dependent on genomic context (e.g. intergenic vs exonic). Pook et al. (2020) used local CNNs (LCNNs), which define region-specific filters, to accommodate this biological feature. Although comparisons with classical method were mixed, LCNNs were the best DL architecture considered (Pook et al. 2020). Second, differences in the computational resources needed to train different models and their long-term predictive stability are under-considered. Research on the long-term performance of DL models in simulated breeding programs and efficiency-normalized prediction accuracies would fill knowledge gaps in the possible advantages and disadvantages of DL models for breeding. Third, differences in the metrics (Pearson's *r* and RMSE) used for evaluation of GP and DL models are not sufficiently appreciated. Arguably, correlation is the more relevant metric for breeding applications, where the objective is most often prediction of the ranks of selection candidates. An alternative metric that may better fit existing DL pipelines is to optimize the prediction of pairwise differences between selection candidates as proposed by Piepho (1998). Overall, more nuanced evaluation of the sources of error in predictions and their relevance to the intended application could guide model development and improvement by revealing where potential for improvement exists.

Finally, replications of past results and comparison with previous models are hampered by three disciplinary differences between the fields of quantitative genetics and DL. First, DL researchers benchmark models on standardized datasets such as MNIST or CIFAR10. For GP problems, a dataset on 599 wheat lines in 4 environments collected by CIMMYT and distributed with the BGLR R package has informally served as a benchmark dataset. However, it does not possess the scale and complexity required to address new prediction problems. Standardized, publicly available datasets of sufficient scale and complexity would serve as valuable baselines for the comparison of different classical models and DL architectures. A curated dataset from the Genomes to Fields Initiative encompassing 78,686 phenotypic records from 4,372 maize hybrids grown in 136 location-years (Lopez-Cruz et al. 2023) is an example of a dataset that meets these requirements. However, because the performance of GP models also relies on population structure, marker density, and linkage disequilibrium, which differ across breeding populations and crop species, additional datasets with different characteristics are needed. Second, the effort required for hyperparameter optimization can be prohibitive for replicating published models from descriptions of the architectures. Furthermore, training a new model using identical input data and hyperparameters is highly dependent on random initialization of the weights. This could be alleviated by including tables of hyperparameters in publications and depositing trained model weights in public repositories. This would improve the ease of comparing models, especially for researchers with limited computational resources. Third, DL researchers typically evaluate models by their performance on a single train/test split rather than cross-validation as in the GP literature. Given the historical sizes of datasets in both fields, there are good arguments for either approach. Moving forward, it would be beneficial to consider the possible consequences of different evaluation metrics and practices, especially for comparison with the historical literature.

In conclusion, this study outlined three theoretical reasons for the observed empirical differences in the performance of FFNs and classical (gBLUP and RKHS) models for GP problems: capacity to incorporate diverse data modalities, lack of manual feature engineering, and universal approximation properties. We confirmed these theoretical predictions through systematic comparisons between models using a large, complex dataset with extensive hyperparameter tuning that were enabled by combining recent advances in kernel engineering and algorithm development. First, we demonstrated that feature engineering of SNP data from inbred parents or hybrids by PCA reduces prediction accuracy across almost all models tested. Second, we proposed a taxonomy of prediction problems based on available data modalities and the predictand. By applying this taxonomy, we systematically and fairly compared the predictive accuracy of FFNs and RKHS models on five different prediction problems and showed that, for matched prediction problems, RKHS consistently outperforms FFNs. Finally, we summarized findings and trends in the literature applying DL to GP and proposed several methodological improvements and avenues for future research.

For now, classical models, especially RKHS, remain state-of-the-art for GP in plant breeding. Considering the challenges faced by agriculture in the next century, further research into applications of DL to prediction problems in plant breeding should continue and will be enhanced by improvements in theoretical and practical rigor.

## Supplementary Material

jkaf314_Supplementary_Data

## Data Availability

Phenotypic, genomic PCs, soil, and weather and management data are available at 10.5281/zenodo.6916775 (Kick 2022). Hybrid SNPs are available at 10.6084/m9.figshare.22776806 (Lopez-Cruz et al. 2023). Hyperparameter optimization results, trained model weights, and code are available at 10.5281/zenodo.17088040. Code is also available at https://github.com/amkusmec/FeedforwardNetworks-and-GenomicPrediction. Supplemental material available at G3 online.
