# Supplementary material for: Critical evaluation of the theory and practice of feed-forward neural networks for genomic prediction
Source: G3 (Bethesda). 2025 Dec 24;16(3):jkaf314. doi: 10.1093/g3journal/jkaf314 (PMC12958802; doi:10.1093/g3journal/jkaf314)
Supplement: jkaf314_Supplementary_Data [file jkaf314_supplementary_data.pdf]

Supplementary figures

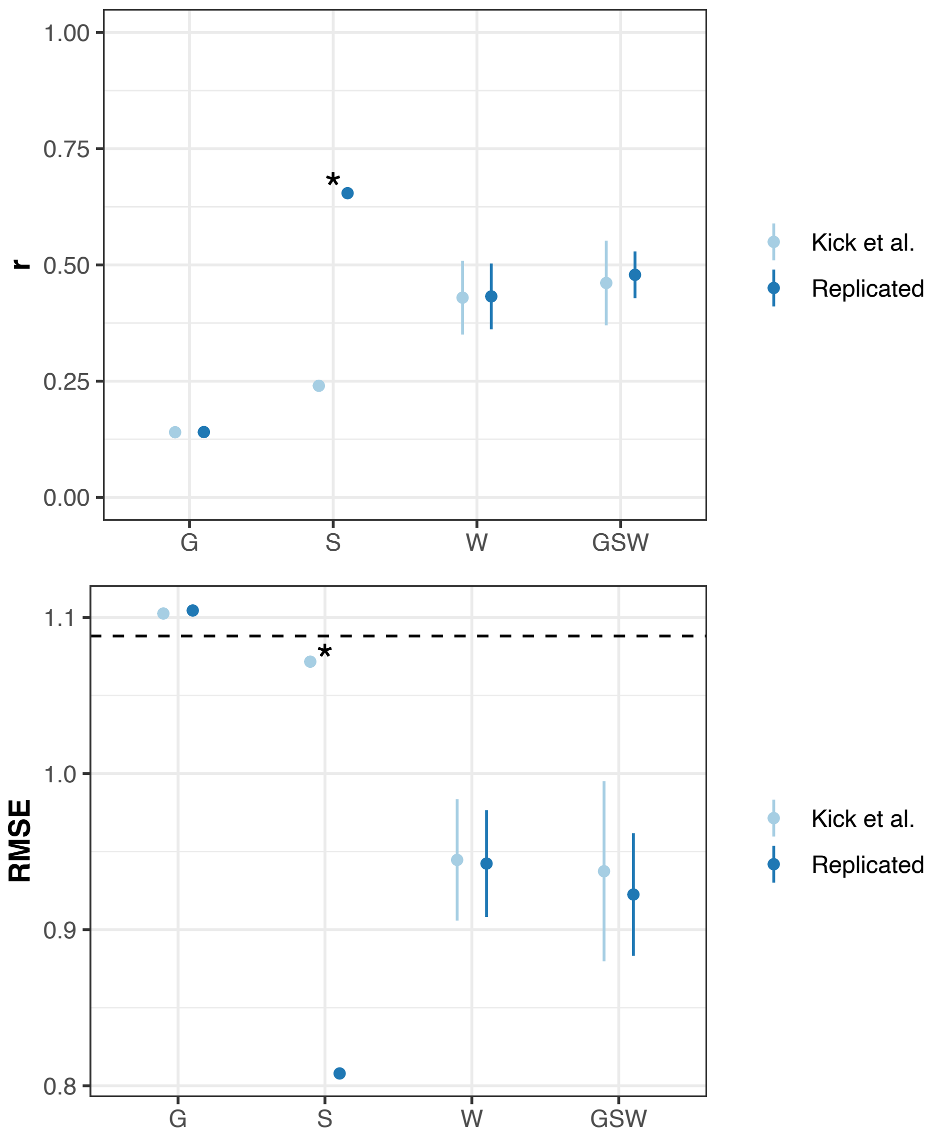

Figure S1. Replicated prediction accuracy of linear kernel/GBLUP models. Accuracy is measured by Pearson's r or root mean square error (RMSE) on an independent test set. Points indicate the mean of 10 replicates with different random seeds; vertical lines, ±1 standard deviation. Horizontal lines indicate 0 (r) or RMSE for an intercept-only model. "*" indicates $p \leq 0.05$ for a two-sample t-test of unequal means. "G" indicates the use of genomic PCs only. "S" indicates the use of soil data only. "W" indicates the use of weather data only. "GSW" indicates the use of genomic PCs, soil, and weather data and two-way interactions with genomic PCs.

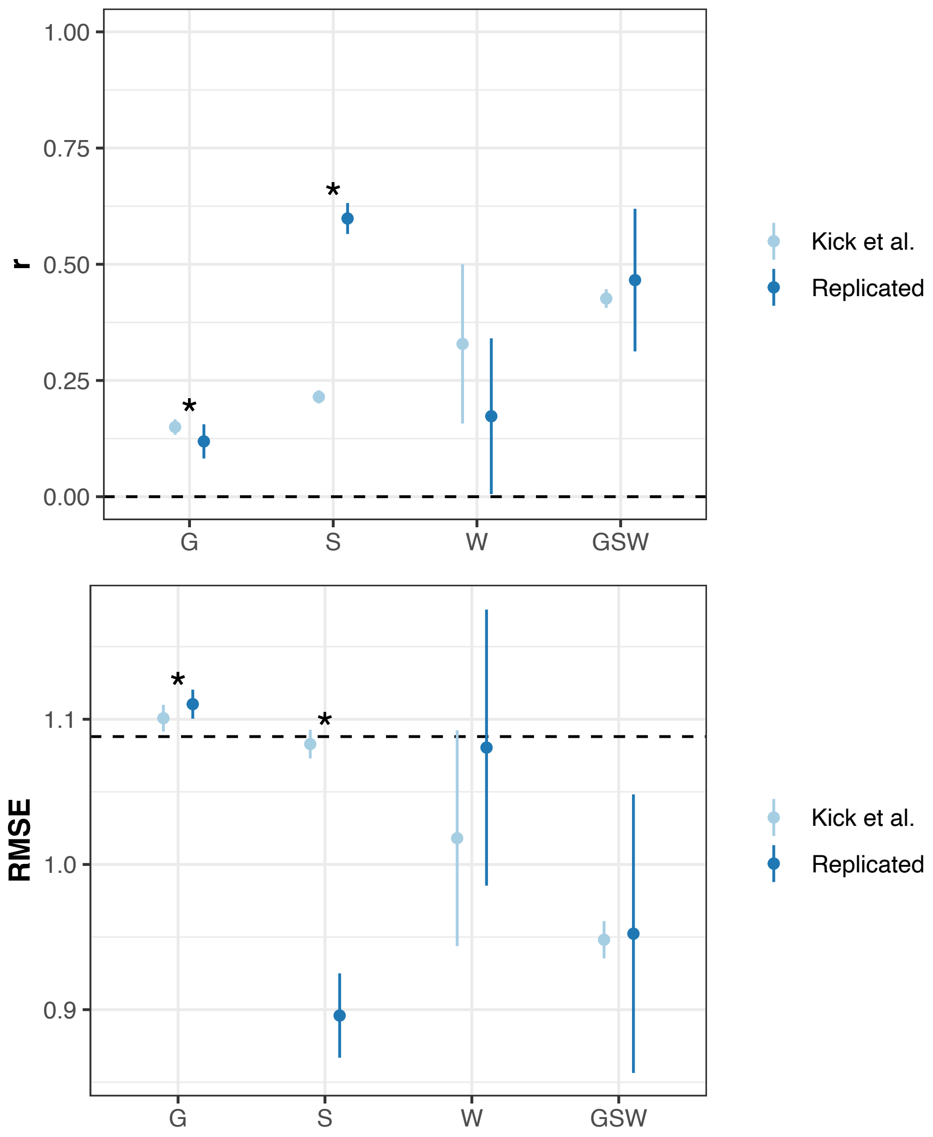

Figure S2. Replicated prediction accuracy of neural networks. Accuracy is measured by Pearson's r or root mean square error (RMSE) on an independent test set. Points indicate the mean of 10 replicates with different random seeds; vertical lines, ±1 standard deviation. Horizontal lines indicate 0 (r) or RMSE for an intercept-only model. "*" indicates $p \leq 0.05$ for a two-sample t-test of unequal means. "G" indicates the use of genomic PCs only. "S" indicates the use of soil data only. "W" indicates the use of weather data only. "GSW" indicates the use of genomic PCs, soil, and weather data and two-way interactions with genomic PCs.

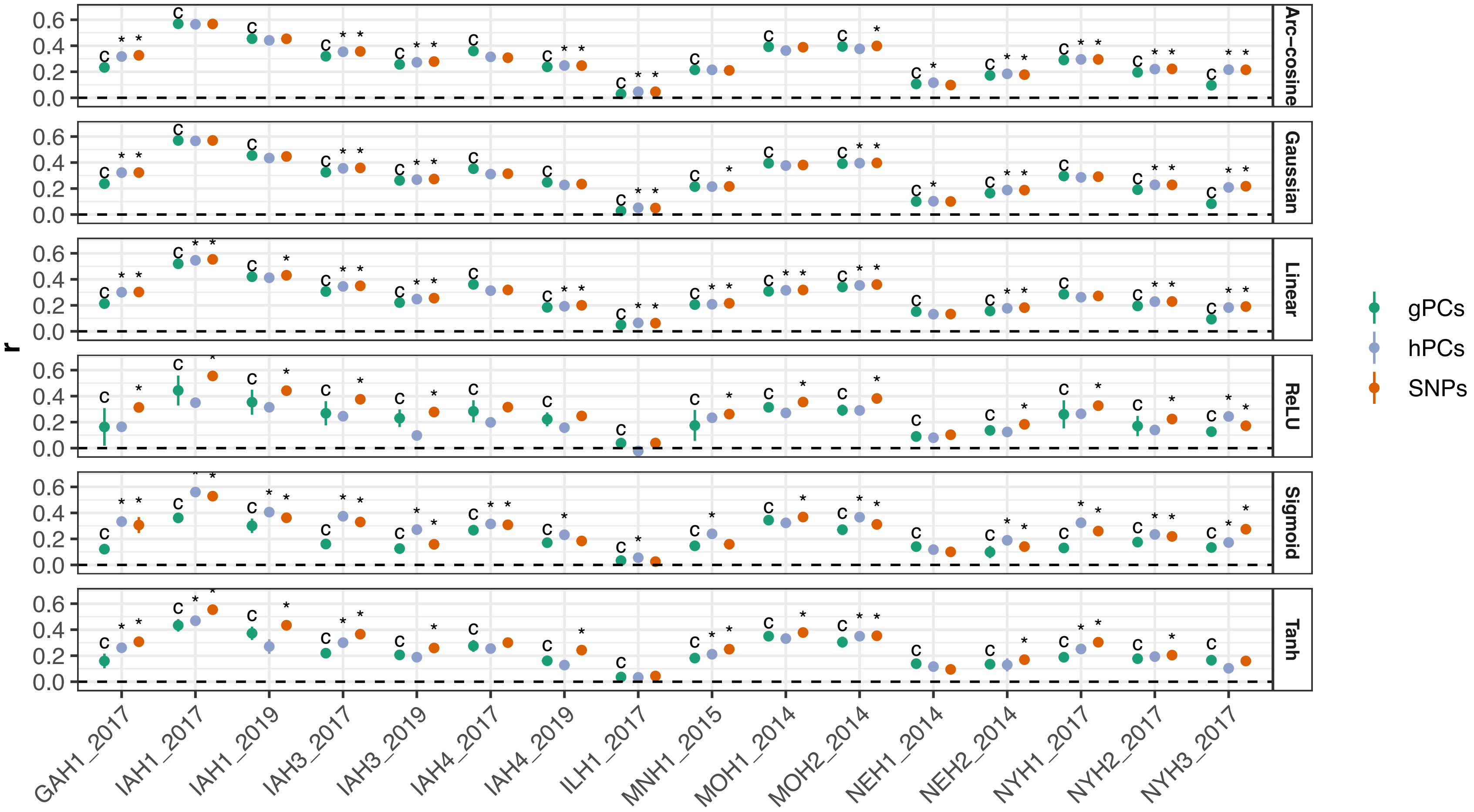

Figure S3. Within-environment prediction accuracy using genomic PCs, hybrid PCs, or SNPs as inputs. Accuracy is measured by Pearson's r on an independent test set. Points indicate the mean of 10 replicates with different random seeds; vertical lines, ±3 standard errors of the mean. Horizontal lines indicate $r = 0$. "*" indicates $p \leq 0.05$ for Dunnett's test against the control ("c") condition that Pearson's r is larger between genomic data types within each model class and environment.

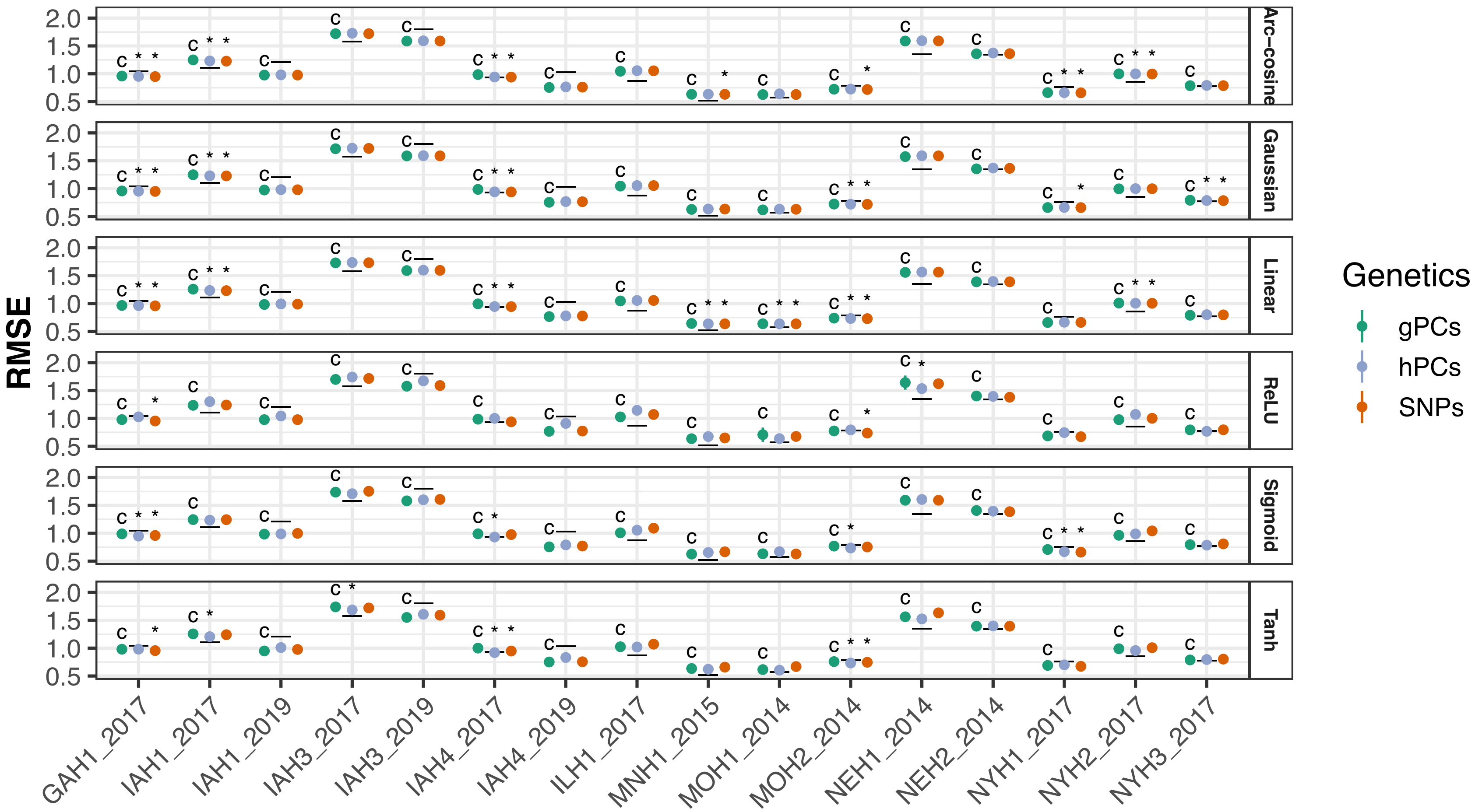

Figure S4. Within-environment prediction accuracy using genomic PCs, hybrid PCs, or SNPs as inputs. Accuracy is measured by root mean square error (RMSE) on an independent test set. Points indicate the mean of 10 replicates with different random seeds; vertical lines, ±3 standard errors of the mean. Horizontal lines indicate the RMSE for an intercept-only model. "*" indicates $p \leq 0.05$ for Dunnett's test against the control ("c") condition that RMSE is smaller between genomic data types within each model class and environment.

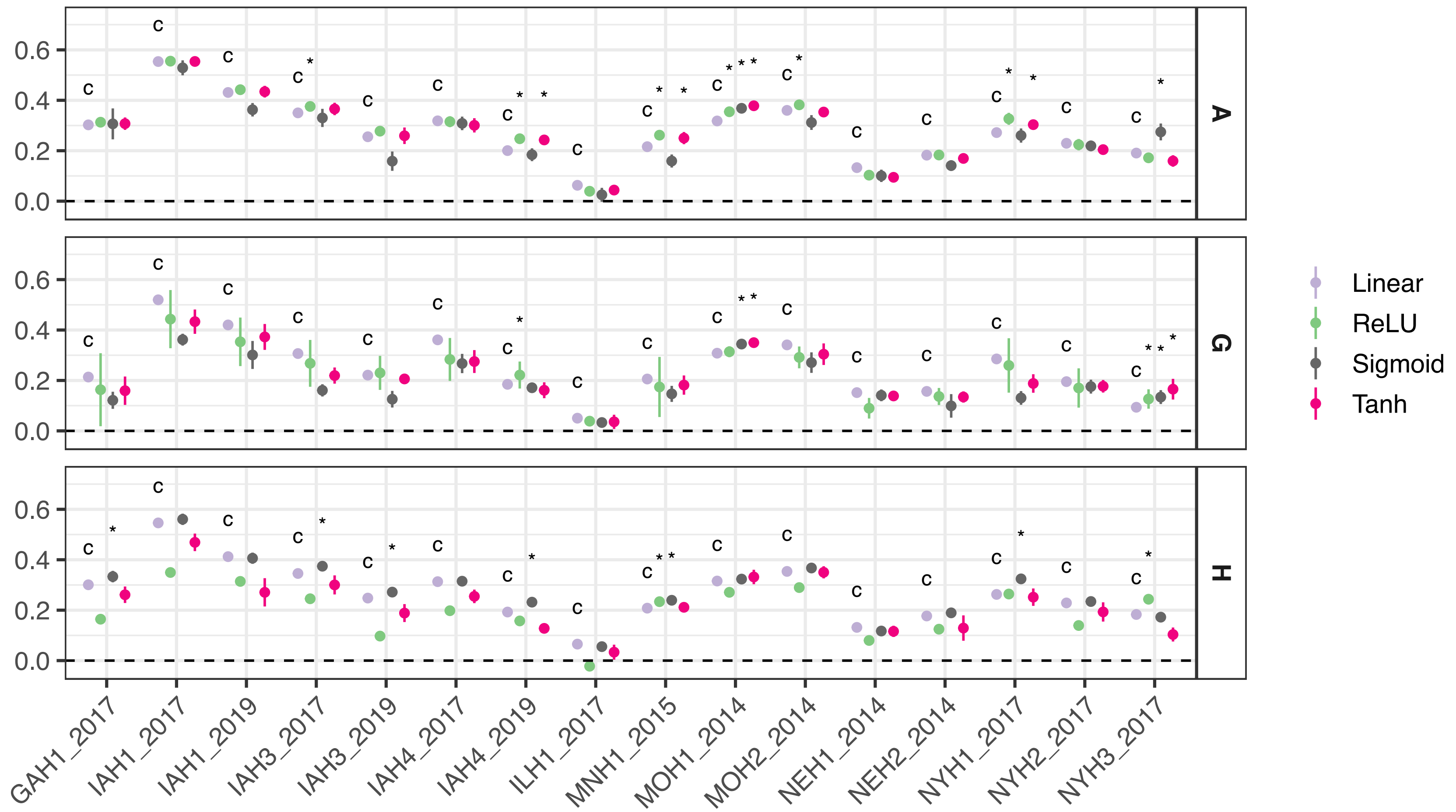

Figure S5. Within-environment breeding value prediction accuracy using a linear kernel (RKHS) or neural networks. Accuracy is measured by Pearson's r on an independent test set. Points indicate the mean of 10 replicates with different random seeds; vertical lines, ±3 standard errors of the mean. Horizontal lines indicate $r = 0$. "*" indicates $p \leq 0.05$ for Dunnett's test against the control ("c") condition that Pearson's r is larger for different model classes within each data type and environment. "G" indicates the use of genomic PCs only; "H", the use of hybrid PCs only; and "A", the use of additive genetic effects only. "SW" indicates the use of soil and weather data and two-way interactions with the genetic data.

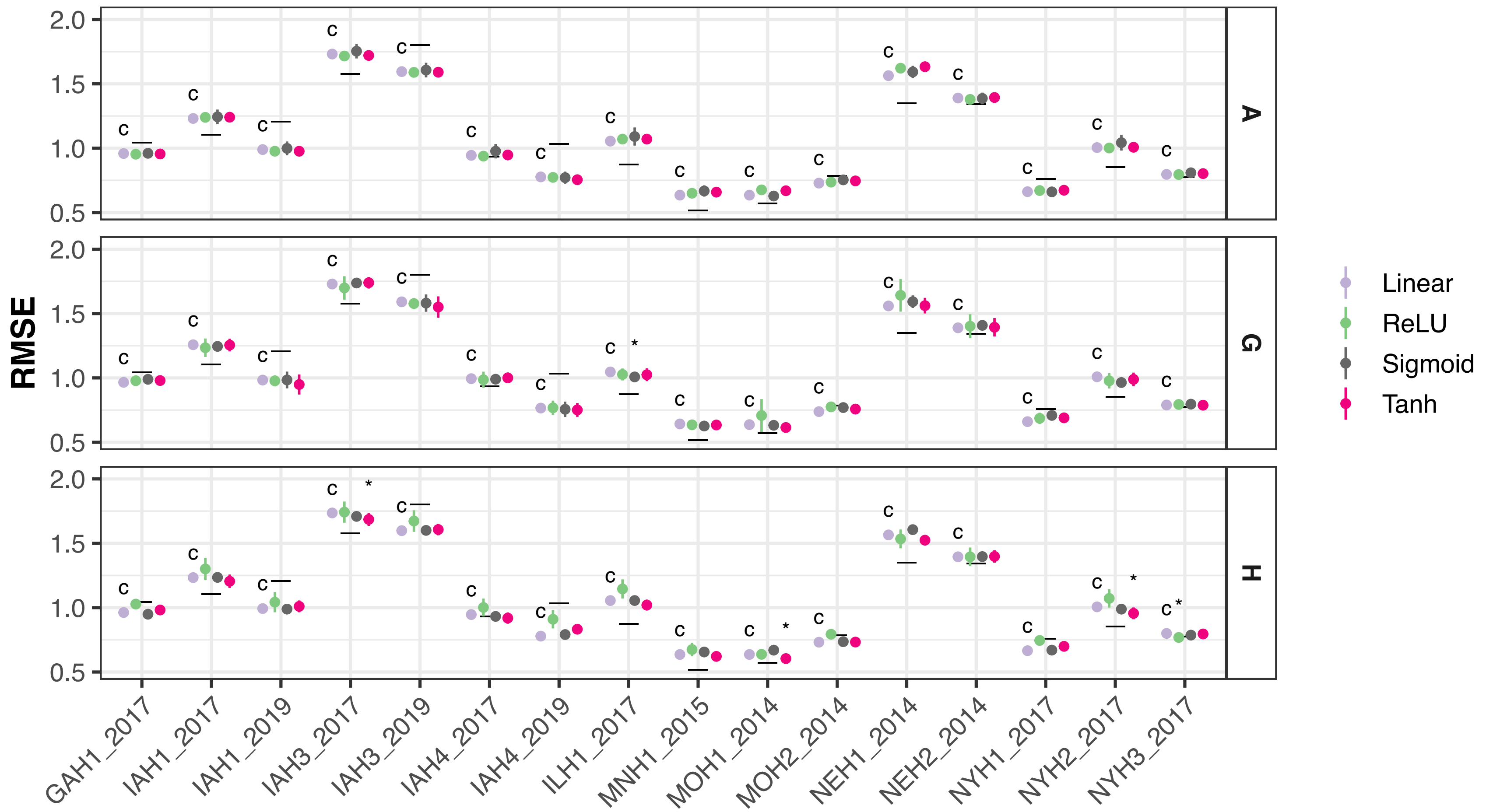

Figure S6. Within-environment breeding value prediction accuracy using a linear kernel (RKHS) or neural networks. Accuracy is measured by root mean square error (RMSE) on an independent test set. Points indicate the mean of 10 replicates with different random seeds; vertical lines, ±3 standard errors of the mean. Horizontal lines indicate RMSE for an intercept-only model. "*" indicates $p \leq 0.05$ for Dunnett's test against the control ("c") condition that RMSE is smaller for different model classes within each data type and environment. "G" indicates the use of genomic PCs only; "H", the use of hybrid PCs only; and "A", the use of additive genetic effects only. "SW" indicates the use of soil and weather data and two-way interactions with the genetic data.

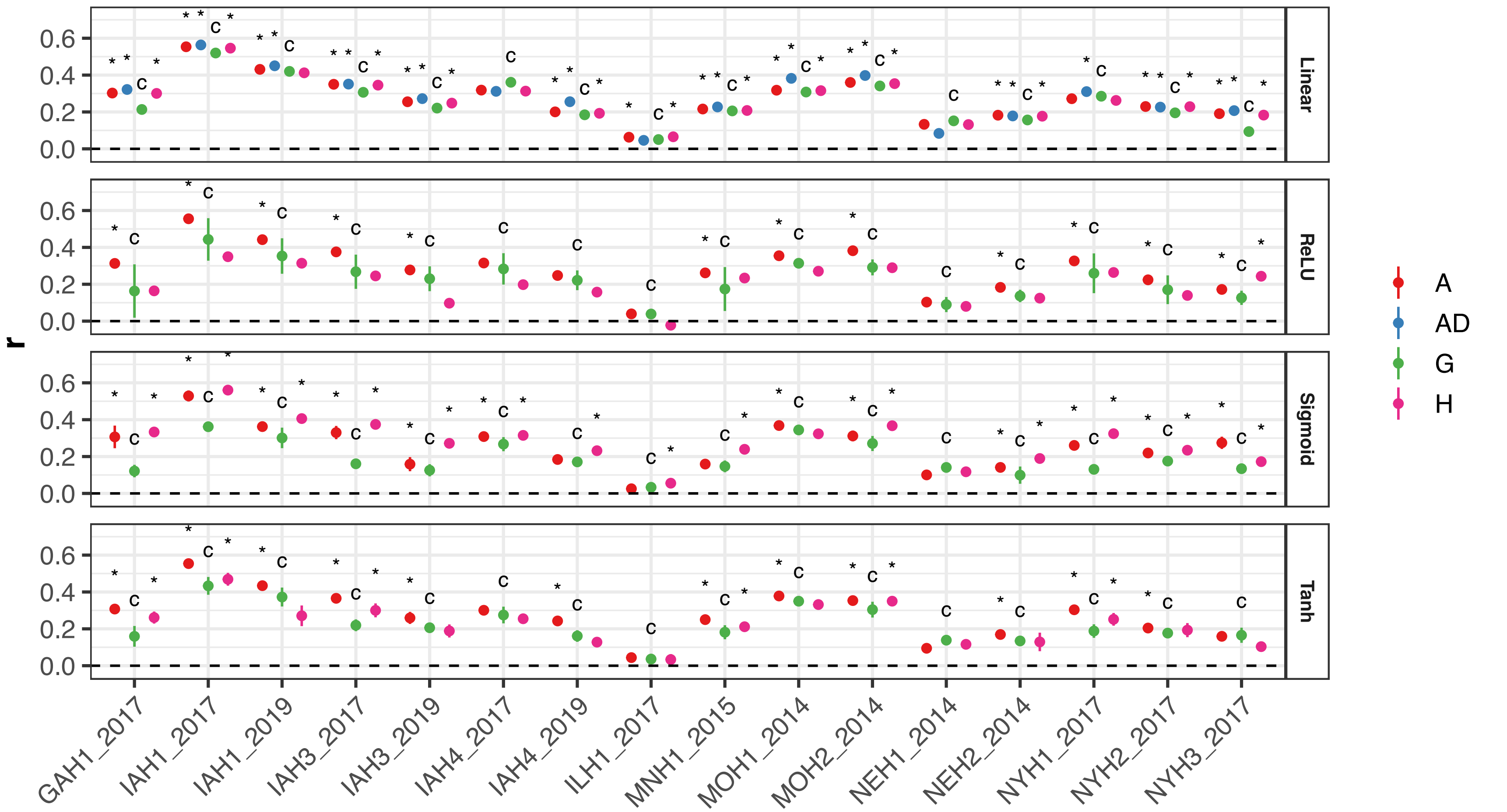

Figure S7. Within-environment hybrid prediction accuracy using RKHS models or neural networks. Accuracy is measured by Pearson's r on an independent test set. Points indicate the mean of 10 replicates with different random seeds; vertical lines, ±3 standard errors of the mean. Horizontal lines indicate $r = 0$. "*" indicates $p \leq 0.05$ for Dunnett's test against the control ("c") condition that Pearson's r is larger for different data types within each model class and environment. "G" indicates the use of genomic PCs; "H", the use of hybrid PCs; "A", the use of additive genetic effects only; and "AD", the use of dominance deviations.

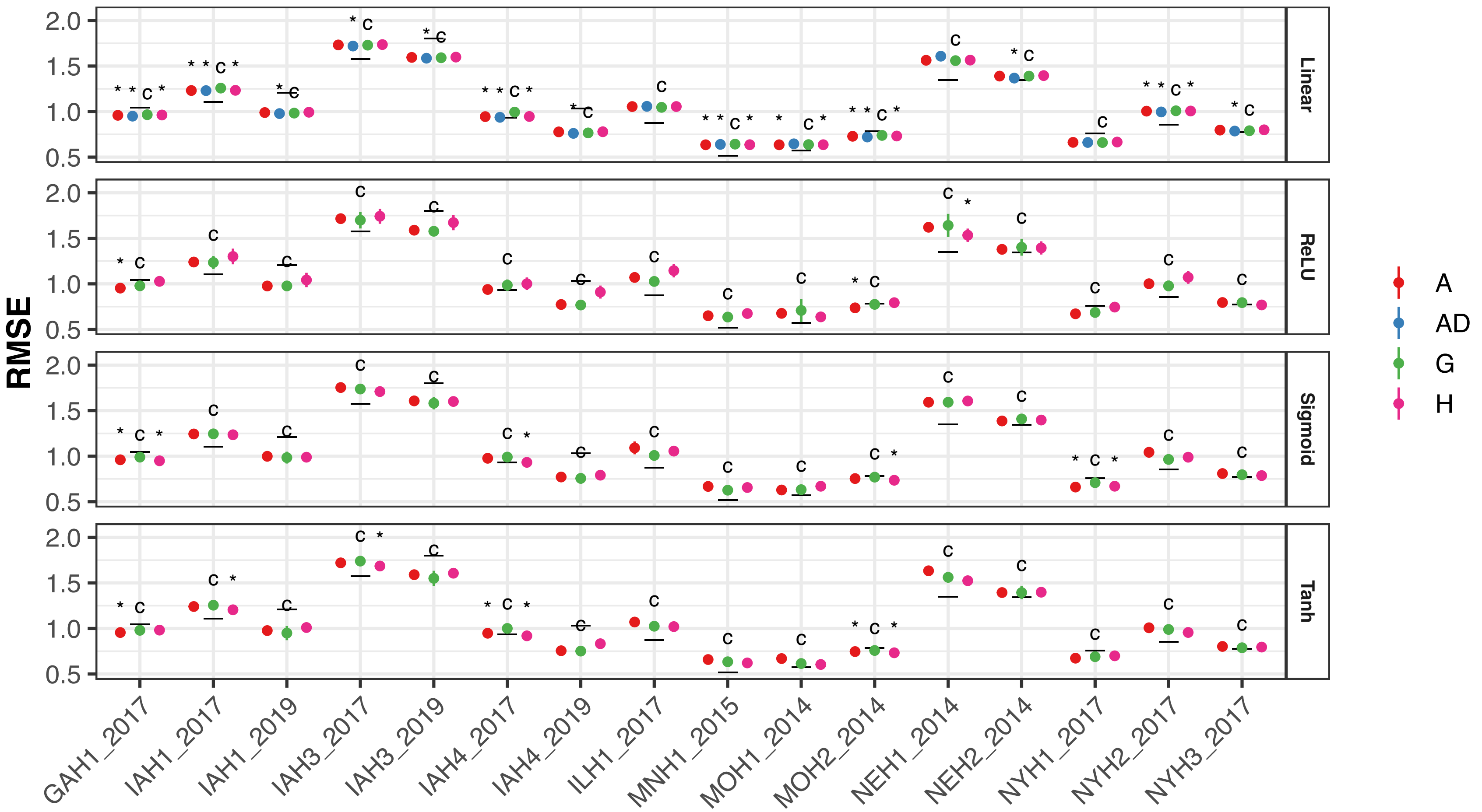

Figure S8. Within-environment hybrid prediction accuracy using RKHS models or neural networks. Accuracy is measured by root mean square error (RMSE) on an independent test set. Points indicate the mean of 10 replicates with different random seeds; vertical lines, ±3 standard errors of the mean. Horizontal lines indicate RMSE for an intercept-only model. "*" indicates $p \leq 0.05$ for Dunnett's test against the control ("c") condition that RMSE is smaller for different data types within each model class and environment. "G" indicates the use of genomic PCs; "H", the use of hybrid PCs; "A", the use of additive genetic effects only; and "AD", the use of dominance deviations.

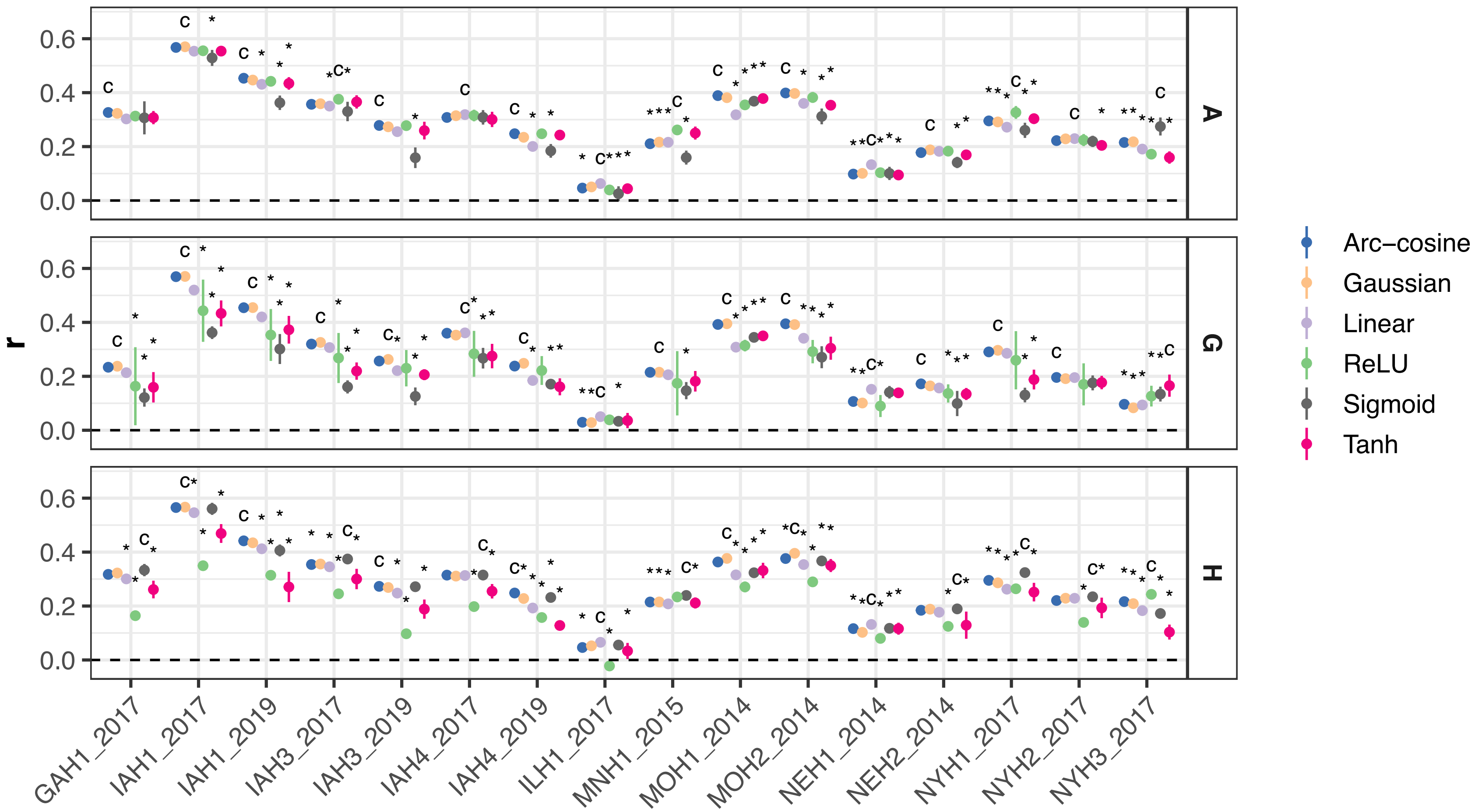

Figure S9. Within-environment total genetic value prediction accuracy using RKHS models or neural networks. Accuracy is measured by Pearson's r on an independent test set. Points indicate the mean of 10 replicates with different random seeds; vertical lines, ±3 standard errors of the mean. Horizontal lines indicate $r = 0$. "*" indicates $p \leq 0.05$ for Dunnett's test against the control ("c") condition that Pearson's r is smaller for different model classes within each data type and environment. The control condition is chosen to be the model with the largest average Pearson's r. "G" indicates the use of genomic PCs only; "H", the use of hybrid PCs only; and "A", the use of additive genetic effects only.

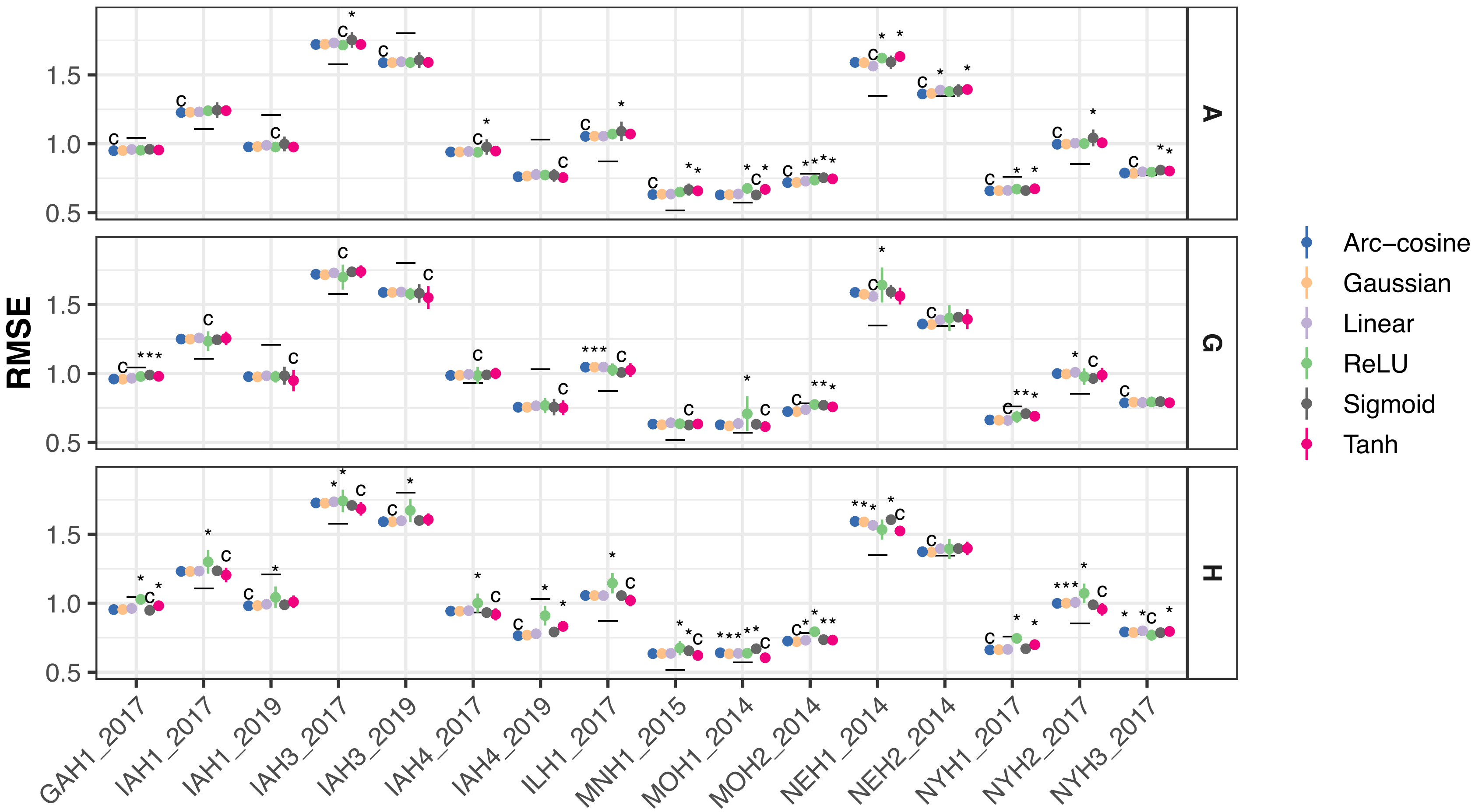

Figure S10. Within-environment total genetic value prediction accuracy using RKHS models or neural networks. Accuracy is measured by root mean square error (RMSE) on an independent test set. Points indicate the mean of 10 replicates with different random seeds; vertical lines, ± 3 standard errors of the mean. Horizontal lines indicate RMSE for an intercept-only model. "*" indicates $p \leq 0.05$ for Dunnett's test against the control ("c") condition that RMSE is larger for different model classes within each data type and environment. The control condition is chosen to be the model class with the smallest average RMSE. "G" indicates the use of genomic PCs only; "H", the use of hybrid PCs only; and "A", the use of additive genetic effects only.

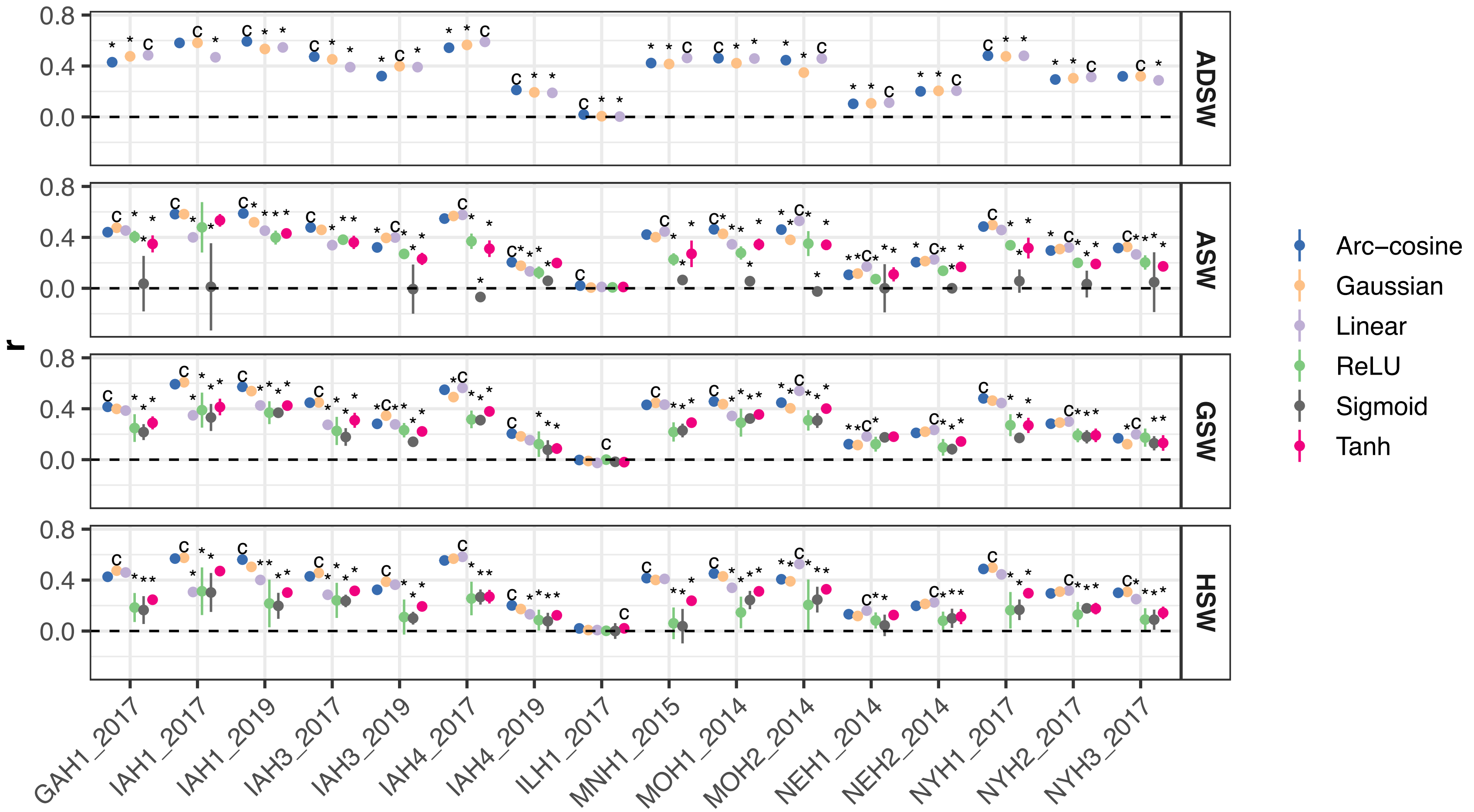

Figure S11. Within-environment phenotype prediction accuracy using RKHS models or neural networks. Accuracy is measured by Pearson's r on an independent test set. Points indicate the mean of 10 replicates with different random seeds; vertical lines, ±3 standard errors of the mean. Horizontal lines indicate $r = 0$. "*" indicates $p \leq 0.05$ for Dunnett's test against the control ("c") condition that Pearson's r is smaller for different model classes within each data type and environment. The control condition is chosen to be the model class with the largest average Pearson's r. "G" indicates the use of genomic PCs; "H", the use of hybrid PCs; "A", the use of additive genetic effects only; and "AD", the use of dominance deviations. All models use soil ("S") and weather ("W") data and their two-way interactions with the genetic data.

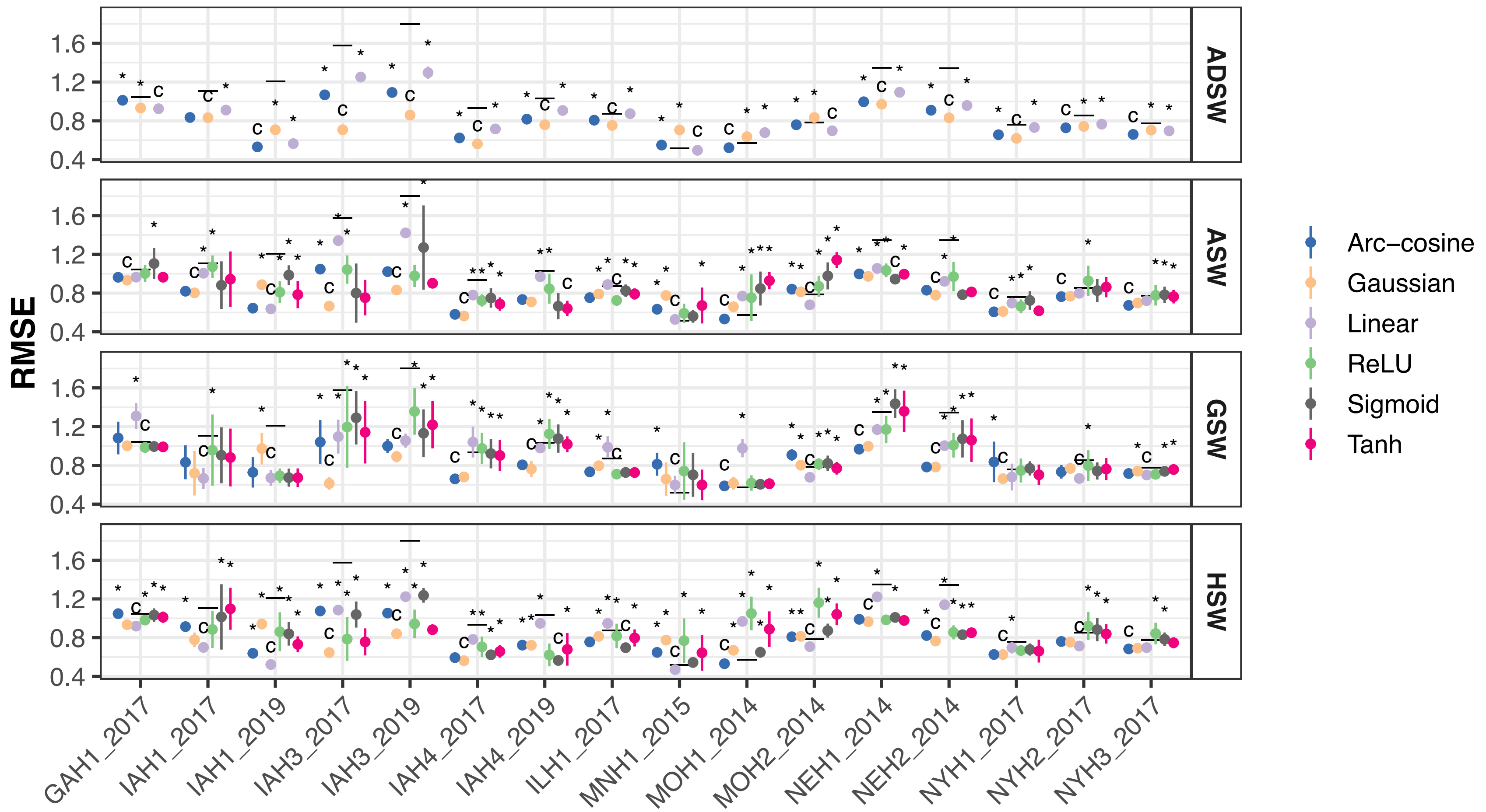

Figure S12. Within-environment phenotypic prediction accuracy using RKHS models or neural networks. Accuracy is measured by root mean square error (RMSE) on an independent test set. Points indicate the mean of 10 replicates with different random seeds; vertical lines, ±3 standard errors of the mean. Horizontal lines indicate RMSE for an intercept-only model. "*" indicates $p \leq 0.05$ for Dunnett's test against the control ("c") condition that RMSE is larger for different model classes within each data type and environment. The control condition is chosen to be the model class with the smallest average RMSE. "G" indicates the use of genomic PCs; "H", the use of hybrid PCs; "A", the use of additive genetic effects only;

and “AD”, the use of dominance deviations. All models use soil (“S”) and weather (“W”) data and their two-way interactions with the genetic data.
